# The malignancy of chordomas is enhanced via a circTLK1/miR-16-5p/Smad3 positive feedback axis

**DOI:** 10.1038/s41420-023-01332-1

**Published:** 2023-02-15

**Authors:** Jingbing Lou, Hongliang Zhang, Qingshan Huang, Chenglong Chen, Wei Wang, Jianfang Niu, Jiuhui Xu, Tingting Ren, Yi Huang, Xiaodong Tang, Wei Guo

**Affiliations:** 1 Musculoskeletal Tumor Center, Peking University People’s Hospital, No. 11 Xizhimen South Street, Beijing, 100044 People’s Republic of China; 2 Beijing Key Laboratory of Musculoskeletal Tumor, No. 11 Xizhimen South Street, Beijing, 100044 People’s Republic of China

**Keywords:** Sarcoma, miRNAs

## Abstract

CircRNAs play crucial roles in various malignancies via an increasing number of reported regulatory mechanisms, including the classic sponging mechanism between circRNAs and micro RNAs (miRNAs). We performed bioinformatic analyses and identified circTLK1 as a regulator of malignant chordoma progression. Moreover, we observed that circTLK1 showed high expression in chordoma cells and tissues, while circTLK1 interference suppressed chordoma cell proliferation and invasion. In addition, circTLK1 directly interacted with miR-16-5p, which has previously been shown to repress chordoma, and circTLK1 knockdown suppressed Smad3 expression. Chromatin immunoprecipitation sequencing further demonstrated that Smad3 acts as a positive regulator by interacting with *TLK1*, thereby mediating the circTLK1/miR-16-5p/Smad3 positive feedback axis. Taken together, our findings suggested that the disruption of the circTLK1/miR-16-5p/Smad3 positive feedback pathway, particularly via the Smad3 inhibitor SIS3, could be a promising therapeutic strategy.

## Introduction

Chordomas are predominantly located in the spine and cranial basement [1, 2]. Despite having a low incidence (approximately 0.8 patients of one million individuals), chordomas have an alarmingly high rate of recurrence [3] and often do not respond to conventional chemotherapy, which is problematic for researchers and clinical physicians [4, 5]. Consequently, complete surgical resection combined with radiotherapy remain the preferred standard treatment approach [5]. However, a few studies have investigated the mechanisms of initiation, progression, and drug resistance in chordomas.

To date, the molecular pathogenesis of chordomas have been reported to involve impaired brachyury (*TBXT*) expression, other aberrant genes, and non-coding RNAs (ncRNAs) [6, 7]. However, the study about circRNAs in the chordoma is relatively scarce. Specially, circRNAs are a new type of ncRNAs that were originally thought to be the “garbage byproducts” of precursor RNA splicing [8]. A variety of detected circRNAs exert crucial effects via sponging regulation, protein translation, and transcriptional regulation [9, 10]. For instance, circRNAs can sponge miRNAs to regulate the initiation or proliferation of malignancies such as colorectal cancer, breast cancer, pancreatic ductal adenocarcinoma, and osteosarcoma [11–14]. In particular, the circRNA CCDC66 was found to promote colon cancer [15], while circPRKCI can promote the growth of lung adenocarcinoma [16] and circ0008399 can promote the m6A methyltransferase complex assembly and activity in bladder cancer [17].

MiRNAs are one of the most important downstream targets that can be sponged by circRNAs. Given that miRNAs interact with the 3′-untranslated region of mRNAs, known as the seed sequence, to suppress their translation, regulatory networks involving circRNAs, miRNAs, and mRNAs could exert considerable effects in various diseases, including malignant cancers [18, 19]. For instance, the miR-181a regulating SFRP4 has been shown in ovarian cancer [20]. Previously, we found that miR-16-5p inhibited chordoma growth and progression via suppressing Smad3 expression [21]. Therefore, having identified circTLK1 which sponge miR-16-5p using starBase v3.0, we further explored its oncogenic effects in chordomas and demonstrated that circTLK1 mediates a positive feedback loop.

## Results

### Identification of circTLK1 and its features in chordoma

**Fig. 1:**
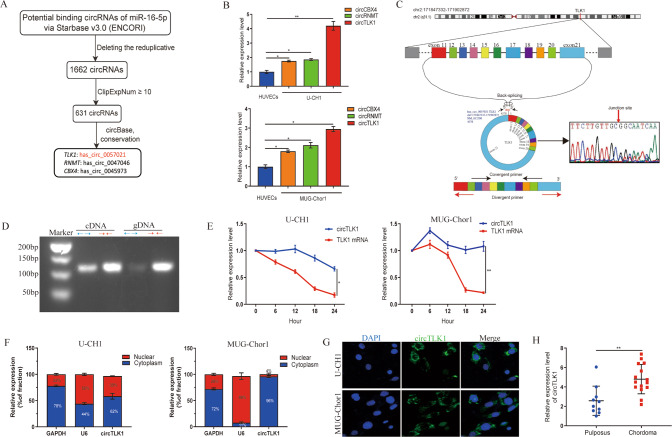
Identification and features of circTLK1 in chordoma. **A** Schematic drawing of circTLK1, circCBX4, and circRNMT screened via Starbase. **B** The relative expression levels of all circRNAs identified in chordoma cells were quantified by RT-qPCR. **C** The circular structure of circTLK1 was illustrated and the junction site was confirmed by Sanger sequencing. **D** The agarose gel electrophoresis result showed circTLK1 amplification from cDNA and not gDNA. **E** The stability of circTLK1 was verified via actinomycin D treatment. **F** The cellular location of circTLK1 was confirmed by nuclear and cytoplasmic RNA extraction combing with RT-qPCR. **G** RNA-FISH illustrated the cellular location of circTLK1. **H** The expression of circTLK1 in chordoma tissues (*n* = 15) vs pulposus (*n* = 10) was quantified via RT-qPCR. *P < 0.05, **P < 0.01, ns indicates no significance.

First, we performed bioinformatic analyses based on targetSites, bioComplex and clipReadNum criterion in the starBase v3.0 database and identified three circRNAs targeting miR-16-5p, namely, circTLK1, circCBX4, and circRNMT (Fig. 1A). RT-qPCR showed that circTLK1 was upregulated to a greater degree than other circRNAs in chordoma cells (Fig. 1B).

CircTLK1 (circBase ID: hsa_circ_0057021) is composed of exons 11–21 of *TLK1* by post-transcriptional back-splicing. Further, we verified the junction site of circTLK1 using Sanger sequencing (Fig. 1C). Since the circular structure may be produced via back-splicing or gene rearrangement, we attempted to confirm its most probable origin by performing RT-qPCR with divergent and convergent primers on cDNA and gDNA extracted from U-CH1 cells. Interestingly, these results showed that circTLK1 was only present in cDNA, and not in gDNA (Fig. 1D). Furthermore, we confirmed that circTLK1 was relatively stable than TLK1 mRNA after actinomycin D treatment (Fig. 1E). The quantity of cytoplasmic RNA demonstrated that circTLK1 existed significantly in the cytoplasm but not nuclei (Fig. 1F). The cellular location of circTLK1 was further confirmed via RNA-fluorescence in situ hybridization (FISH), which showed a dramatic cytoplasmic orientation (Fig. 1G). CircTLK1 was also highly expressed in chordoma tissues (Fig. 1H). Taken together, these findings confirmed the identical characteristics of circTLK1 and its high expression level in chordoma.

### CircTLK1 promotes the malignancy of chordoma cell in vitro

**Fig. 2:**
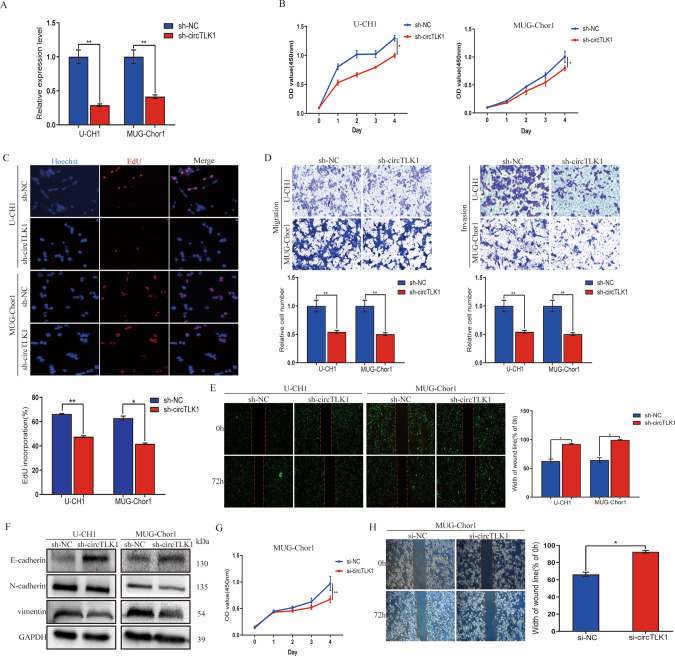
CircTLK1 promotes the malignancy of chordoma cell in vitro. **A** The efficacy of transfection was verified RT-qPCR. **B** CCK8 results showed the viability of U-CH1 and MUG-Chor1 cells interfered by sh-circTLK1. **C** The ability of proliferation was confirmed by EdU assay after silencing circTLK1. **D** Migration and invasion ability were assessed by Transwell assays after silencing circTLK1. **E** Wound-healing assay result showed the migration of chordoma cells with circTLK1 interference. **F** Western blotting results showed the expression level of E-cadherin, N-cadherin, and vimentin proteins with knocked out circTLK1. **G** CCK8 results showed the decrease of MUG-Chor1 cell viability after transfecting another siRNA. **H** The mobility of MUG-Chor1 cell transfected with si-circTLK1 was confirmed by wound-healing assay. *P < 0.05, **P < 0.01.

Loss-of-function assays were performed by constructing circTLK1-knockout vector. RT-qPCR analysis confirmed that the lentiviral vectors containing sh-circTLK1 substantially interfered with circTLK1 in chordoma cells (Fig. 2A). Notably, circTLK1 interference suppressed chordoma cells proliferation, as confirmed using CCK8 (Fig. 2B), and 5-ethynyl-2′-deoxyuridine (EdU) assay (Fig. 2C). CircTLK1 interference reduced the ability of mobility and invasion (Fig. 2D), as further confirmed by scratch assay (Fig. 2E).

To further elucidate the relationship between circTLK1 and epithelial-mesenchymal transition (EMT), we performed western blotting analysis, which showed that circTLK1 interference promoted E-cadherin, but suppressed N-cadherin and vimentin (Fig. 2F). In addition, to avoid the off-target effects on circTLK1 interference, another si-circTLK1 was involved. The proliferation of chordoma cells was then inhibited after interfering circTLK1 (Fig. 2G). The motility of chordoma cells was inhibited after interfering circTLK1(Fig. 2H). In conclusion, above results suggest that circTLK1 advances the malignancy of chordoma cells.

### MiR-16-5p suppresses chordoma malignancy via interacting with Smad3

**Fig. 3:**
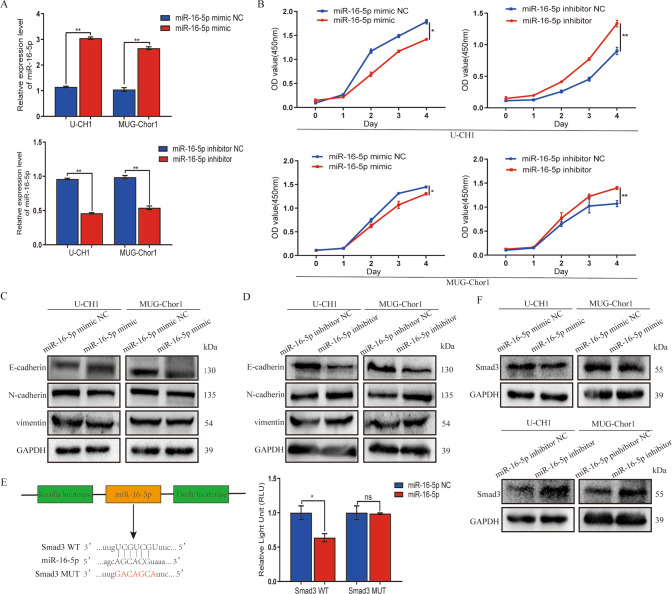
MiR-16-5p suppresses chordoma malignancy via interacting with Smad3. **A** The expression level of miR-16-5p was quantified by RT-qPCR in chordoma cells with a miR-16-5p mimic or inhibitor. **B** CCK8 results indicated the viability in the miR-16-5p mimic or inhibitor group. **C**, **D** The expression levels of E-cadherin, N-cadherin, and vimentin proteins were quantified using western blotting. **E** The interaction between miR-16-5p and Smad3 was verified by a dual-luciferase reporter gene assay. **F** The expression level of Smad3 protein was quantified by western blot. *P < 0.05, **P < 0.01, ns indicates no significance.

Based on our previous findings, next, miR-16-5p was interfered and overexpressed. RT-qPCR results showed that the expression level increased in the overexpression group but that of interference group was opposite (Fig. 3A). Subsequent CCK8 demonstrated that chordoma cell viability was reduced by the mimics but promoted by the inhibitors (Fig. 3B).

Since EMT progression is of vital importance in chordoma malignancy, related proteins were quantified. All results showed that N-cadherin and vimentin expression deceased but E-cadherin expression increased in overexpression group (Fig. 3C), whereas the interference group exerted the opposite effect (Fig. 3D). The relative intensity of luciferase decreased in wild-type vector (Fig. 3E). Smad3 expression was inhibited by the mimics but promoted by the inhibitors (Fig. 3F), confirming that miR-16-5p interacts with Smad3 to suppress chordoma cell malignancy.

### CircTLK1 interacts with miR-16-5p to enhance Smad3 expression

**Fig. 4:**
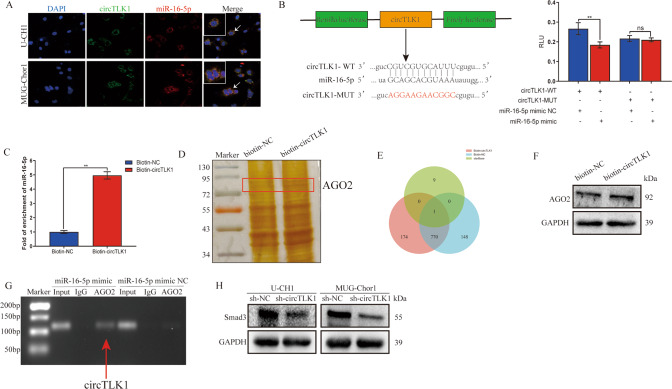
CircTLK1 interacts with miR-16-5p to enhance Smad3 expression. **A** FISH images showed the cellular location of circTLK1 and miR-16-5p. **B** The target site of the combination was confirmed by a dual-luciferase reporter gene assay. **C** The interaction between circTLK1 and miR-16-5p was verified using RNA pull-down assay. **D** Silver staining demonstrated the difference of target proteins between biotin-circTLK1 and biotin-NC. **E** The Venn diagram showed that specific protein between circTLK1 and miR-16-5p were identified by mass spectrum combining with starBase prediction. **F** AGO2 protein was assessed by western blot between biotin-circTLK1 and biotin-NC. **G** RIP assay illustrated the interaction between circTLK1 and miR-16-5p. **H** The expression level of Smad3 was verified by western blot after transfecting sh-circTLK1. **P < 0.01, ns indicates no significance.

Based on FISH findings of circTLK1 and miR-16-5p, we observed that both predominantly located in the chordoma cellular cytoplasm (Fig. 4A). Luciferase findings revealed the deletion of relative luciferase intensity in the circTLK1-WT group (Fig. 4B). RNA pull down assay indicated that circTLK1 was enriched when biotinylated bead complexes were pulled down (Fig. 4C). Further, proteins combining with circTLK1/miR-16-5p were eluted from pulled down biotinylated bead complexes. The specific interacted protein was identified via silver staining combined with mass spectrum. The results showed that AGO2 protein was the specific functional protein as a mediator between circTLK1 and miR-16-5p (Fig. 4D, E). Western blotting also demonstrated the increased AGO2 protein expression in the biotin-circTLK1 group (Fig. 4F). Next, we used anti-AGO2 antibody to immunoprecipitate the complex produced by RNA immunoprecipitation (RIP) assay, combined with agarose gel electrophoresis illustrating that circTLK1 enrichment was significantly much more in the miR-16-5p mimic group (Fig. 4G). Based on these results, circTLK1/miR-16-5p/Smad3 axis was further verified by western blot, which showed a significantly decreased Smad3 expression level in the sh-circTLK1 group (Fig. 4H). Thus, circTLK1 targets with miR-16-5p to regulate Smad3 and exert oncogenic effects.

### Smad3 interacts with *TLK1* to regulate circTLK1 transcription via a positive feedback loop

**Fig. 5:**
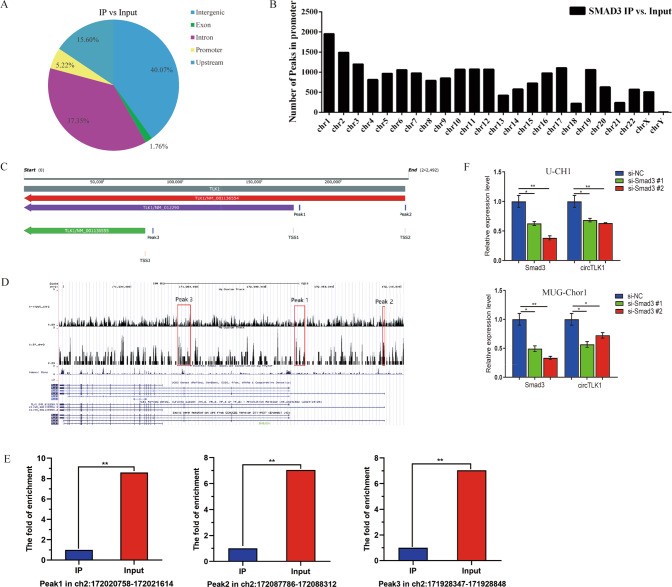
Smad3 interacts with the *TLK1* promoter. **A** The different fractions of genes bound by Smad3 was illustrated. **B** The chromosome distribution of ChIP peaks was shown. **C**–**E** Three peaks in chromosome 2 bound by Smad3 were shown. **F** The expression level of circTLK1 was quantified by RT-qPCR with si-Smad3 interference. *P < 0.05, **P < 0.01.

To clarify the target of Smad3, chromatin immunoprecipitation sequencing (ChIP-seq) was involved. The schematic diagram showed that there are 5.22% DNA fractions located in the promoter region of genes bound by Smad3 (Fig. 5A). The number of the peaks located in promoters with over 6-fold enrichment (*p* < 1*e*^−20^, FDR < 0.01) distributed on every chromosome were counted. It shown that Smad3 combined with chromosomes 1, 2, and 3 with the highest combination (Fig. 5B). There were three peaks observed in the *TLK1* gene region of all 20, 371 sequencing results (*p* < 1*e*^−^^20^, FDR < 0.01; Table 1). Based on the ChIP-seq results, three peaks in the upstream and promoter regions of *TLK1* with >6.5-fold enrichment were likely to be Smad3 binding sites (Fig. 5C–E). What’s more, the expression of Smad3 and circTLK1 decreased in chordoma cells after transfecting with si-Smad3＃1 and si-Smad3＃2 (Fig. 5F). In conclusion, Smad3 functions as a positive modulator that promotes the transcription of circTLK1 through a positive feedback pathway.

**Table 1:** The detailed information of 3 peaks in the upstream and promoter region of *TLK1*.

Peak position	*P*-value	Fold enrichment	FDR	Refseq_name	Symbol	TSS	Strand	Peak to TSS
chr2:172020758-172021614	1.53E−09	8.83	0.0024	NM_012290	TLK1	172017403	–	−3782
chr2:172087786-172088312	2.65E−07	6.95	0.0072	NM_001136554	TLK1	172087803	–	−245
chr2:171928347-171928848	3.58E−10	6.95	0.0021	NM_001136555	TLK1	171923483	–	−5114

### CircTLK1 knockdown represses tumor growth

**Fig. 6:**
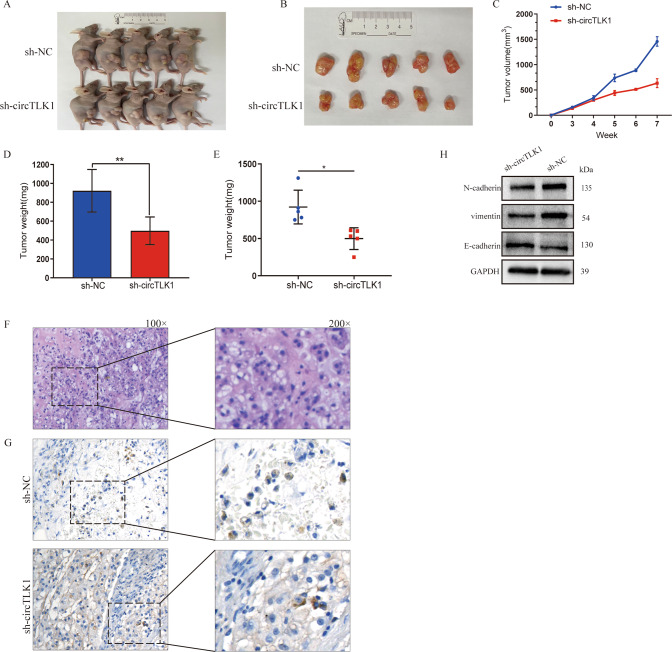
CircTLK1 knockdown represses tumor growth. **A**, **B** The photographs of mice tumors are shown. **C**–**E** The volume and weight of the tumors were analyzed and illustrated. **F**, H & E staining illustrated the pathological characteristics of tumors. **G** Representative image demonstrated Smad3 expression assessed using IHC. **H** Expressions of the proteins E-cadherin, N-cadherin, and vimentin were evaluated using western blotting. *P < 0.05, **P < 0.01.

Finally, we confirmed the effect of circTLK1 by injecting BALB/c nude mice with U-CH1 cells to induce subcutaneous tumors (Fig. 6A, B). They showed decreased tumor mass and volume (Fig. 6C–E). H&E staining of tumors was depicted in Fig. 6F, while Smad3 expression was confirmed using IHC in tumors from sh-circTLK1 or sh-NC tumors (Fig. 6G). Furthermore, we observed decreased EMT progression in sh-circTLK1 tumors (Fig. 6H). Therefore, circTLK1 knockdown suppresses chondroma growth in vivo.

## Discussion

**Fig. 7:**
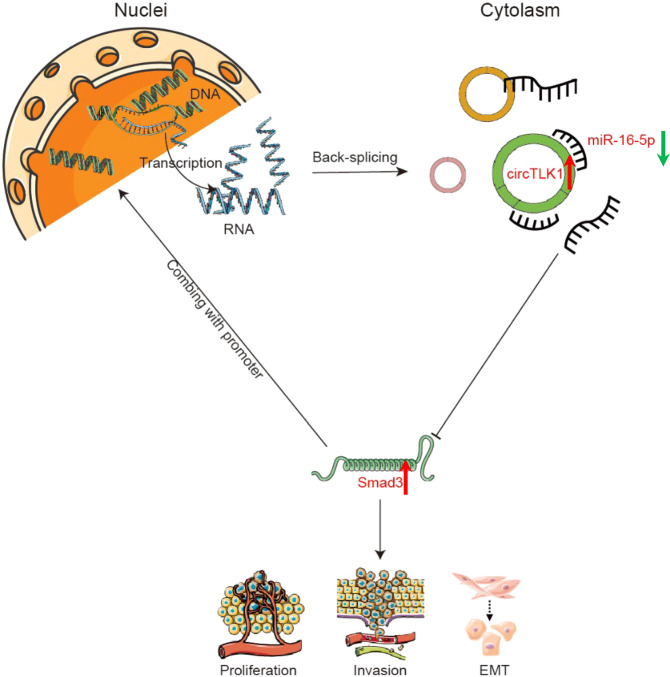
The mechanism illustration of the circTLK1/miR-16-5p/Smad3 pathway is shown. Several diagrams are used to indicate the cellular structure and molecules in our study, such as DNA, mRNA, circRNA, and miRNA. The green arrow (↓) indicates the decreased level, conversely, the red one (↑) is the opposite. The interaction is demonstrated by “→” or “⊣”, which means promotion or suppression, respectively.

CircRNAs participate in various malignant tumors [22–24]. However, the quantity of circRNA obtained in chordomas has been low. Previously, we identified miR-16-5p using miRNA microarrays combined with bioinformatic techniques [21] and predicted circTLK1 to be an upstream target of miR-16-5p using starBase [25]. Traditionally the endogenous ceRNA network, the circRNA-miRNA-mRNA axis, has been explored in diverse scopes, including chemotherapy resistance [26], prognostic biomarker [27], malignancy progression [28], and so on. However, in the present study, we explored the novel mechanism through which circTLK1 promotes chordoma progression by regulating a positive feedback pathway involving miR-16-5p/Smad3 (Fig. 7).

Dysregulated circRNAs sometimes result from mutant genes and oncogenic viruses like EBV [23]. Regretfully, we were unable to elucidate the underlying aberrant molecular mechanism of this circRNA in chordoma. Aberrant *brachyury* is considered as one of the initial factors in chordomas [6], but correlation between *brachyury* and circTLK1 is not investigated in this study. Therefore, although cell function assays demonstrated that aberrant circTLK1 expression was closely associated with chordoma malignancy, the potential mechanism requires further investigation.

Smad3 is a conventional target molecule that has been studied extensively and can be suppressed by miR-16-5p, and that also positively regulates *TLK1* transcription. As a positive mediator, Smad3 bridges the circTLK1/miR-16-5p/Smad3 positive feedback pathway. Moreover, Smad3 has been reported to act as a transcription factor that interacts with several genes [29]. Our findings also revealed that Smad3 integrated with the *TLK1* gene, acting as a transcription factor. Herein, we believe that the Smad3 inhibitor, SIS3, could be used to attenuate the progression of malignant tumors [30]. SIS3 is a kind of selective Smad3 phosphorylation inhibitor. It has been shown to inhibit the phosphorylation of TGF-β/Smad3 in other cancers, such as colorectal cancer [31]. So, it is increasingly significant to verify the effect and value of SIS3 in chordomas. Regrettably, its therapeutic efficacy in chordoma was not involved in the present study warranting further investigation.

CircRNAs were investigated in our study, nevertheless, its clinical significance remains limited. Avenues to disrupt the positive feedback pathway could be explored further. In addition, future studies should aim to elucidate the molecular mechanism underlying chemotherapy resistance to improve the clinical treatment of chondromas. In conclusion, we demonstrated that circTLK1 dysregulation significantly promotes chordoma progression via a positive feedback loop.

## Materials and methods

### Human specimens and cell lines

Chordoma (*n* = 15) and pulposus tissue samples (*n* = 10) were obtained from patients who underwent surgical resection but no chemotherapy in our institution. All of patients have signed informed consent. This study was approved by the Ethics Committee. Chordoma U-CH1 cell line (Cat# CRL-3217) was purchased from ATCC, and MUG-Chor1 initially available at our laboratory was verified via short tandem repeat analysis. Culture flasks were treated according to the instruction of ATCC. Complete Iscove’s modified Dulbecco’s medium (IMDM; ATCC, Cat# 30-2005) and RPMI-1640 (ATCC, Cat# 30-2001; *v/v*, 4:1) were used.

### Isolation of total RNA and gDNA

Total RNA from chordoma cells (U-CH1 or MUG-Chor1) and tissues frozen in liquid nitrogen was isolated using the RNA extraction kit (Qiagen, Germantown, MD, USA, Cat# 74134) and TRIzol reagent. The DNA micro kit (Qiagen, Cat# 56304) was applied to isolate gDNA. The concentration of the extracted RNA was measured using NanoPhotometer Pearl (IMPLEN, Schatzbogen, Munchen, Germany, Model# 03384).

### Nuclear and cytoplasmic RNA extraction

The PARIS™ Kit (Thermo Scientific, Rockford, lL, USA, Cat# AM1921) was used to isolate nuclear and cytoplasmic components. An adequate number of chordoma cells (~10^6^) were lysed in cell fractionation buffer for 5 min. After centrifugation for 3 min, the final nuclear and cytoplasmic RNA were separated and obtained.

### RT-qPCR

All premiers were designed, and RT-qPCR was performed to amplify the target genes. CFX96™ System (Bio-Rad) was used. Intrinsic control such as *GAPDH* as well as *U6*, was used. All primers’ information is presented in Table 2.

**Table 2:** All sequences of premers for target genes.

	Forward (5′→3′)	Reverse (5′→3′)
circTLK1	TGATTGCCGCAACAAGAATGG	ATTGGTAGAGGGTGCCTGAGA
TLK1	GAGTCAGAAATCTTCACATACGTG	AGCCACCTCTACCAAGCAGATG
miR-16-5p	TAGCAGCACGTAAATATTGGCG	TGCGTGTCGTGGAGTC
Smad3	TGAGGCTGTCTACCAGTTGACC	GTGAGGACCTTGTCAAGCCACT
GAPDH	GTCTCCTCTGACTTCAACAGCG	ACCACCCTGTTGCTGTAGCCAA
U6	CTCGCTTCGGCAGCACA	AACGCTTCACGAATTTGCGT

### Agarose gel electrophoresis

The separated circRNAs were confirmed using 2% agarose gel electrophoresis (Shanghai Baygene Biotechnology Company Limited, Shanghai, China, Cat# BY-R0100) and the head-to-tail site of circTLK1 was authenticated via Sanger sequencing with RT-qPCR.

### Actinomycin D treatment

All cells were treated using 100 ng/mL actinomycin D (Merck, Darmstadt, Germany, Cat# SBR00013) at 0, 6, 12, 18, and 24 h. Thereafter, cells were collected to extract total RNA, and the expression of circTLK1 as well as TLK1 mRNA were quantified via RT-qPCR.

### Oligonucleotide, lentivirus, and plasmid transfection

SiRNAs targeting circTLK1 and Smad3 (si-Smad3 #1 and #2), miR-16-5p mimics, and inhibitors were constructed and used to construct sh-circTLK1 or sh-NC lentiviral vectors (Hanbio Biotechnology Company, Shanghai, China). Smad3-overexpression plasmids were purchased from Vigene Biosciences (Shandong, China). All of the cells were transfected with lentivirus supplemented with 0.2% puromycin (Hanbio Biotechnology, Cat# HB-PU-500) for 72 h. Transfection was facilitated with Opti-MEM (Gibco, Cat# 11058021) and Lipofectamine 3000 (Invitrogen, Cat# L3000-015). All the sequences of interference, overexpression, and negative control are listed in Table 3.

**Table 3:** Sequence of shRNA, miRNA mimic, and inhibitor.

		5′→3′
sh-circTLK1		UGCCGCAACAAGAAUGGGUTT
miR-16-5p mimic	Sense	UAGCAGCACGUAAAUAUUGGCG
	Antisense	CCAAUAUUUACGUGCUGCUAUU
miR-16-5p mimic NC	Sense	UUCUCCGAACGUGUCACGUTT
	Antisense	ACGUGACACGUUCGGAGAATT
miR-16-5p inhibitor		CGCCAAUAUUUACGUGCUGCUA
miR-16-5p inhibitor NC		CAGUACUUUUGUGUAUACAA
si-Smad3＃1		GCAACCUGAAGAUCUUCAATT UUGAAGAUCUUCAGGUUGCTT
si-Smad3＃2		GCGUGAAUCCCUACCACUATT UAGUGGUAGGGAUUCACGCTT

### Cell proliferation assays

Briefly, 100 μL of complete IMDM medium and 10 μL of CCK8 reagent (Dojindo Crop, Japan, Cat# CK04) were added to culture plates containing 8 × 10^3^ cells/well. Data were collected over 4 days serially. Chordoma cells were analyzed using an EdU Kit (Beyotime Biotechnology, Beijing, China, Cat# C0075S) to confirm cell proliferation.

### Wound-healing

Chordoma cells were prepared (1 × 10^6^/well) overnight and an artificial wound was scratched. The photographs at the initial and three days were taken under a microscope (Leica Microsystems Inc, Bannockburn, IL, USA), then the width of the wound was measured and analyzed.

### Transwell migration and invasion assays

Transwell chambers (Corning, NY, USA, Cat# 3422) with an 8-μm pore size were used. Specially, 300 μL FBS-free IMDM medium containing 5 × 10^4^ chordoma cells was added up to the top of the chamber. After culturing for 2 days, the following steps were referred to the relevant article [21, 26]. Photographs were taken under the microscope (Leica Microsystems Inc), and migratory cells were counted as well as analyzed.

### Western blotting

Equivalent quantity of protein was added up to 10% SDS-PAGE (Epizyme Biomedical Technology, Shanghai, China, Cat# PG112). Then transferred PVDF membranes (Merk Millipore, Tulagreen, Carrigtwohill, Co Cork IRELAND, Cat# IPVH00010) were blocked with 1× fast-blocking liquid, then incubated with antibodies against Smad3 (1:1000; Cell Signaling Technology, Cat# 9523S), E-cadherin (1:5000, Proteintech, Wuhan, China, Cat# 20874-1-AP), N-cadherin (1:5000; Proteintech, Cat# 22018-1-AP), vimentin (1:5000; Proteintech, Cat# 10366-1-AP), and GAPDH (1:5000; Proteintech, Cat# 10494-1-AP). Specific horseradish peroxidase (HRP)-conjugated secondary antibodies against IgG-mouse or rabbit were hybridized with the primary antibodies in the 37 °C incubator.

### FISH

Firstly, U-CH1 and MUG-Chor1 cells slides were prepared. Then circTLK1 probe and miR-16-5p probe were designed. During preparation, all slides were prepared with being fixed. For pre-hybridization, the slides were incubated at 37 °C. The following steps, hybridization-washing-hybridization, were carried out. Finally, the photographs were taken under a scanning confocal microscope (Leica Microsystems Inc).

### Dual-luciferase gene reporter assay

Both wild and mutant-type vectors were constructed in GenePharma company. In brief, MUG-Chor1 cells transfected with wild and mutant-type circTLK1 or Smad3 were lysed followed by a dual-luciferase assay kit (Beyotime Biotechnology, Cat# RG027). Finally, a microplate reader (Bio-Tek, Santa Clara, CA, USA, Model No. SLXFA) was used to detect relative luciferase intensity.

### IHC staining

Briefly, after deparaffinization, the sections were incubated at 4 °C overnight with corresponding antibodies. The following process was according to the IHC kit (Boster Biological Technology, China, Cat# SA2010). The concentration of Smad3 antibody was used with 1:800. The sections were observed and photos were taken using a Leica microscope.

### RIP

The lysate of MUG-Chor1 cells (~2 × 10^7^) had been prepared with using RIP lysis buffer of EZ-Magna Protein Immunoprecipitation Kit (Millipore, Cat# 17-701), and the lysate were bound to beads-Argonaute 2 (Proteintech, Cat# 10686-1-AP) or IgG antibody complex via gentle agitation overnight at 4 °C.

### RNA pull-down assay

Biotinylated circTLK1 and miR-16-5p were constructed initially, and RNA pull-down assay was performed using Pierce Pull-Down Kit (Thermo Scientific, Cat# 20164). The lysate was agitated gently with M-270 magnetic beads overnight. CircTLK1 and miR-16-5p pull-down were quantified using RT-qPCR. In addition, RNA binding proteins were also purified to identify the specific protein between circTLK1 and miR-16-5p using MS.

### ChIP-seq

A ChIP Kit (Millipore, Cat# 17-610) was applied to chromatin immunoprecipitation (ChIP) assays. In brief, MUG-Chor1 were cross-linked and lysed using lysis buffer. Cellular chromatin was disrupted using ultrasound and then immunoprecipitated using anti-Smad3 antibodies. The target DNA fractions were sequenced and analyzed.

### In vivo animal experiment

The animal experiment was approved by Ethics Committee of our institute. To confirm the oncogenic effect of circTLK1, BALB/c nude mice (*n* = 5 per group) purchased from Beijing Viltalriver were subcutaneously injected with 5 × 10^6^ U-CH1 cells. All mice were separated into different two group at random and blindly. Tumor volume was recorded every week. All mice had been euthanized with the tumor volume not exceeding 2 cm^3^.

### Statistical analysis

Statistical analyses were carried out using SPSS software (v22.0; Armonk, NY, USA) and GraphPad Prism 9.0.0. The method of Student’s *t*-test was involved. No significance was indicated by ns. Significant data are indicated by *P < 0.05, **P < 0.01, ***P < 0.001.

## Supplementary information

original western blot

## Data Availability

The data that were collected and analyzed during the current study are available from the corresponding author on reasonable request.
