# Supplementary material for: The malignancy of chordomas is enhanced via a circTLK1/miR-16-5p/Smad3 positive feedback axis
Source: Cell Death Discov. 2023 Feb 15;9:64. doi: 10.1038/s41420-023-01332-1 (PMC9932141; doi:10.1038/s41420-023-01332-1)

1 ✔Eca1-sh-NC-B,sh-T-B

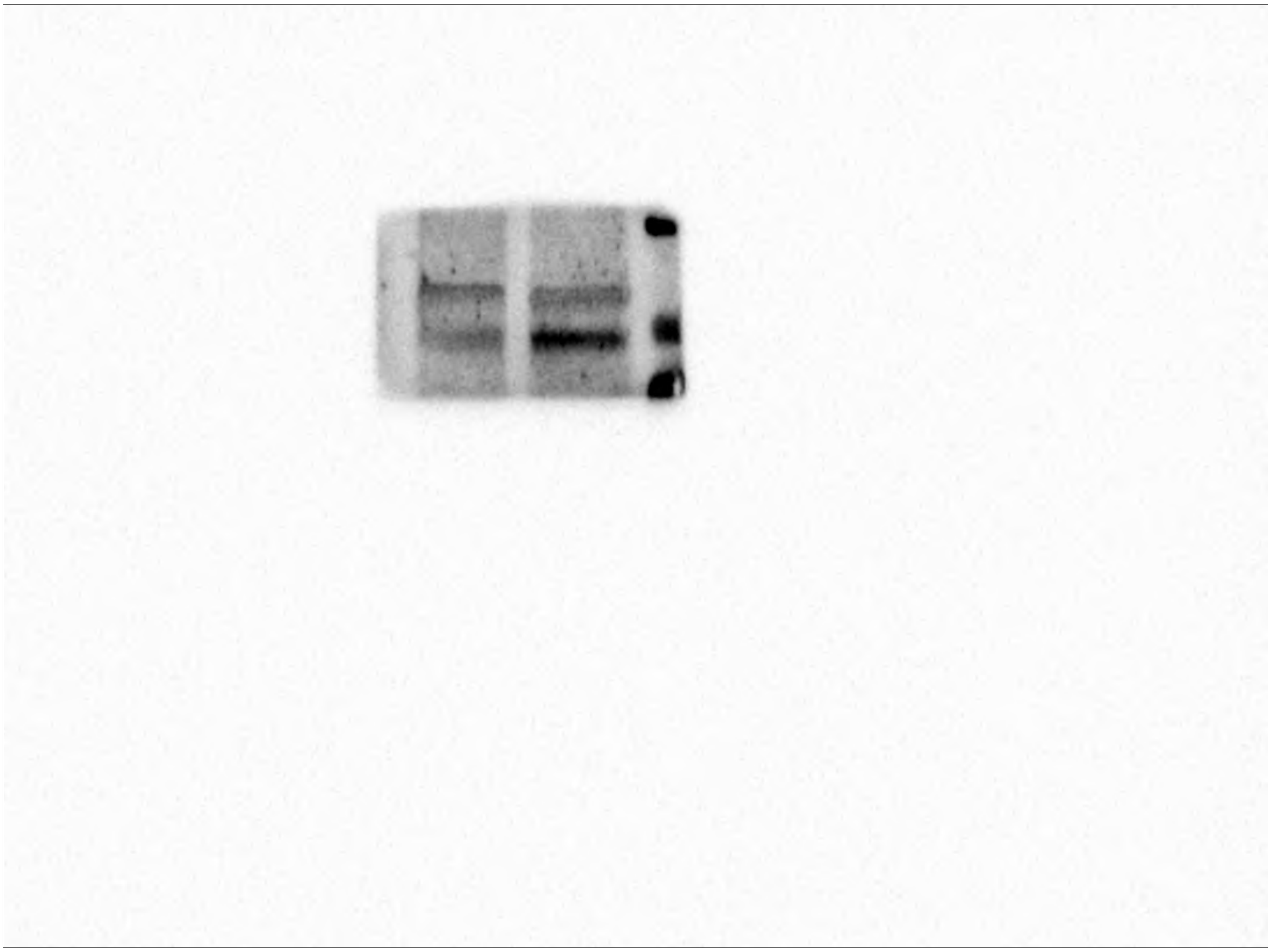

2.✔Nca4.1.1-sh-NC-B,sh-T-B

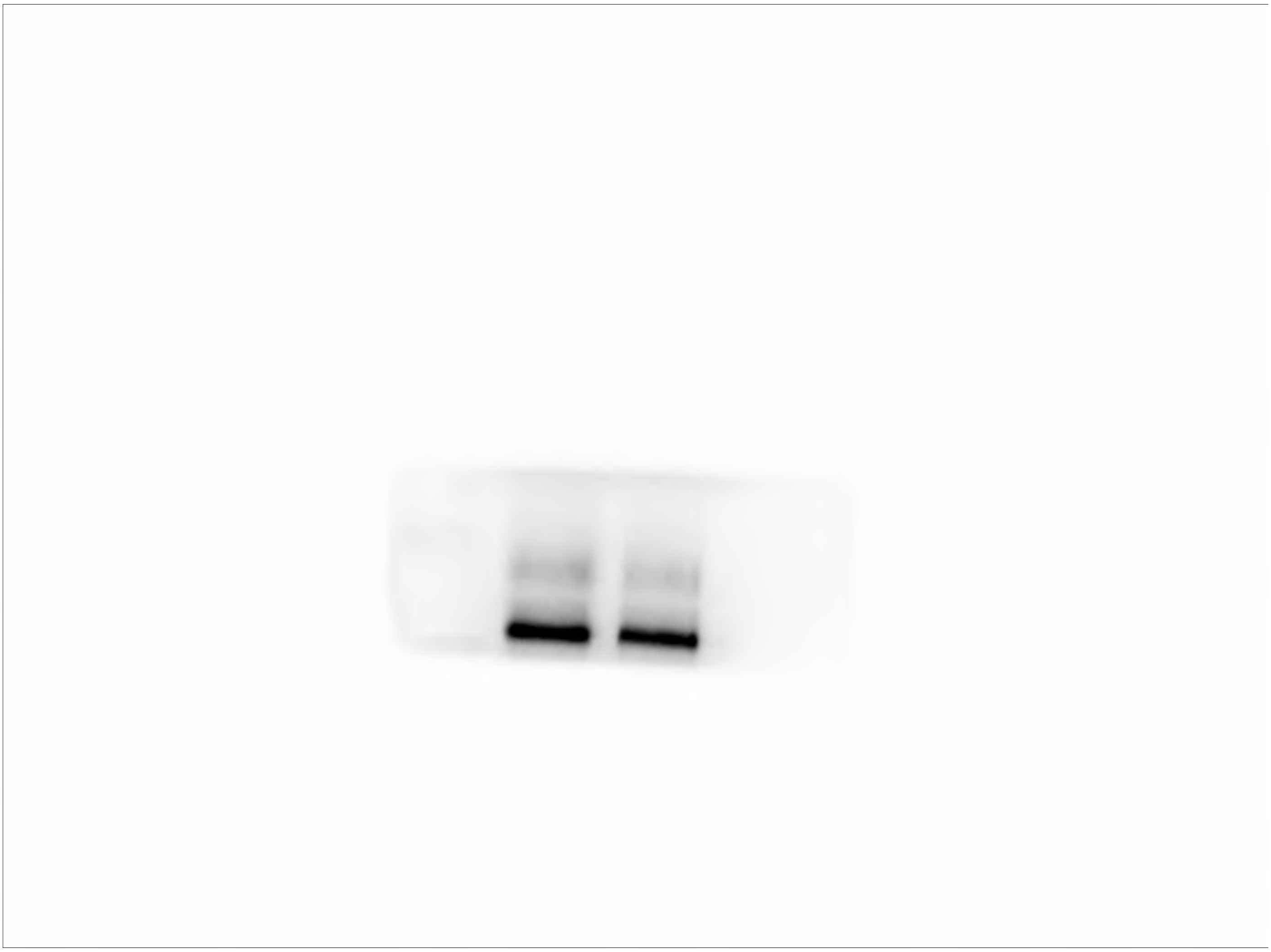

3.Vim-NC mi,16-5p mi,NC in,16-5p in, sh-NC-B,sh-T-B

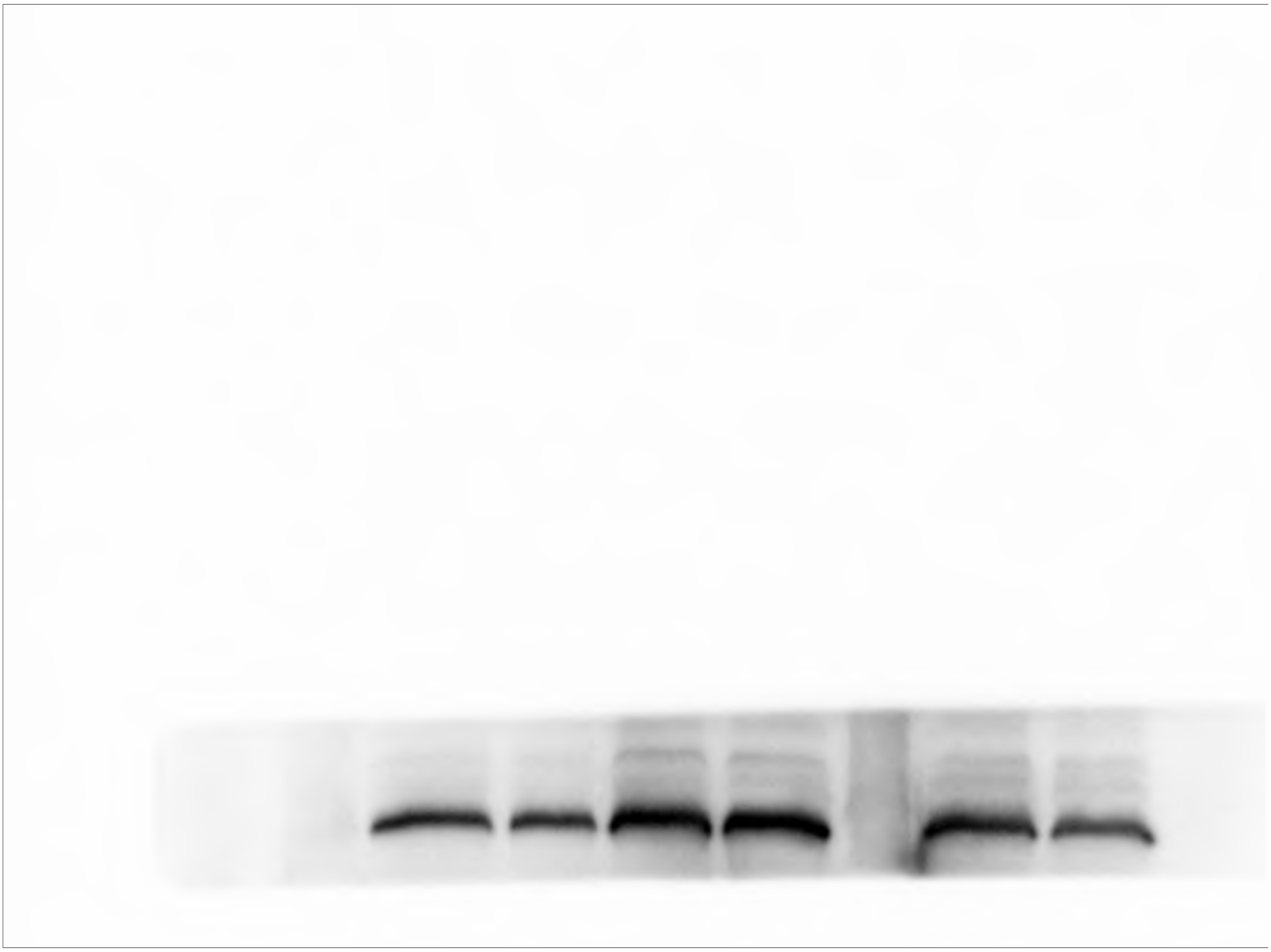

4✔GAPDH5-sh-NC-B,sh-T-B

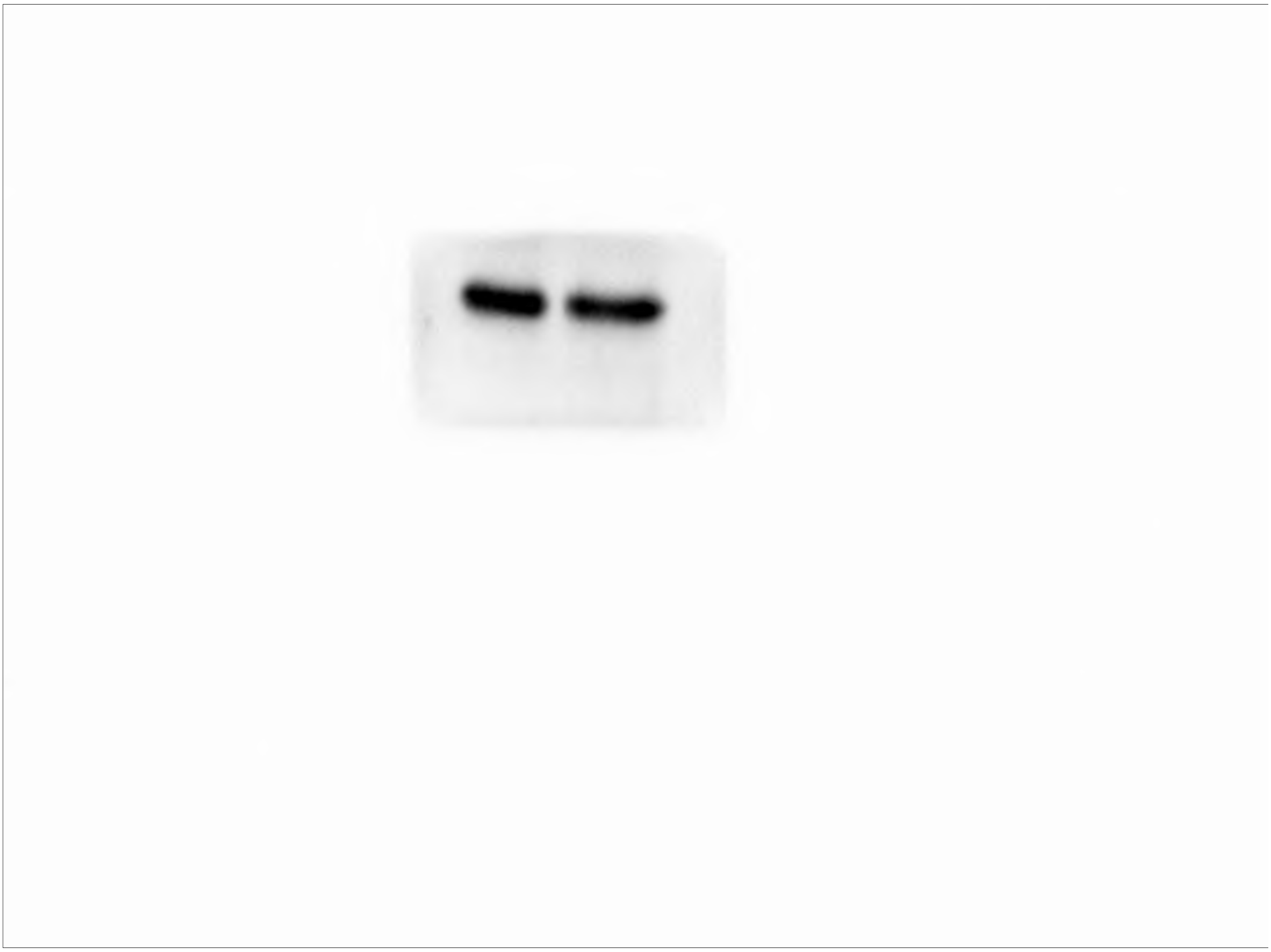

5✔Eca1.1.2-sh-NC-K,sh-T-K

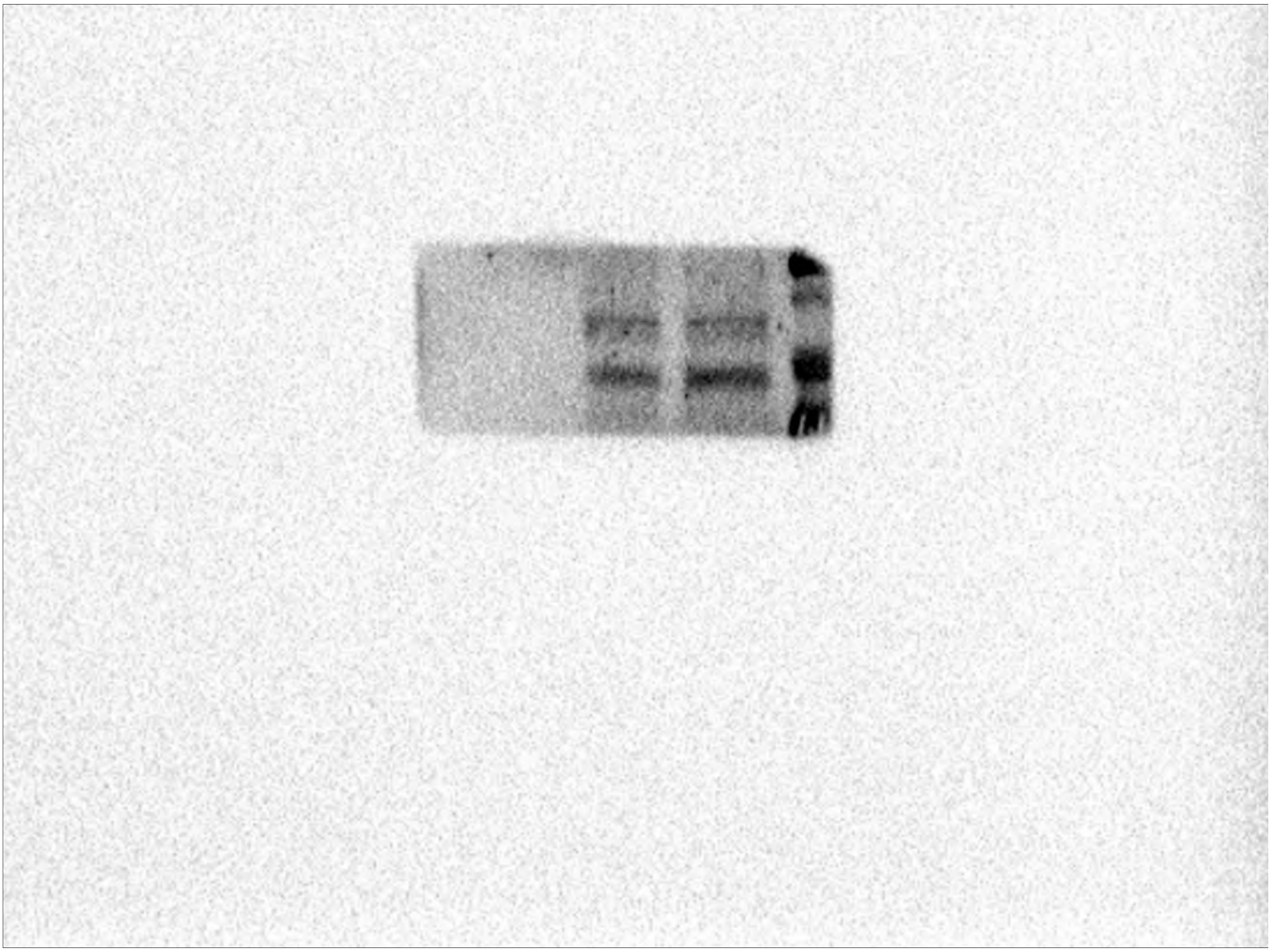

6Nca-NC mi,16-5p mi,NC in,16-5p in, sh-NC-K,sh-T-K,

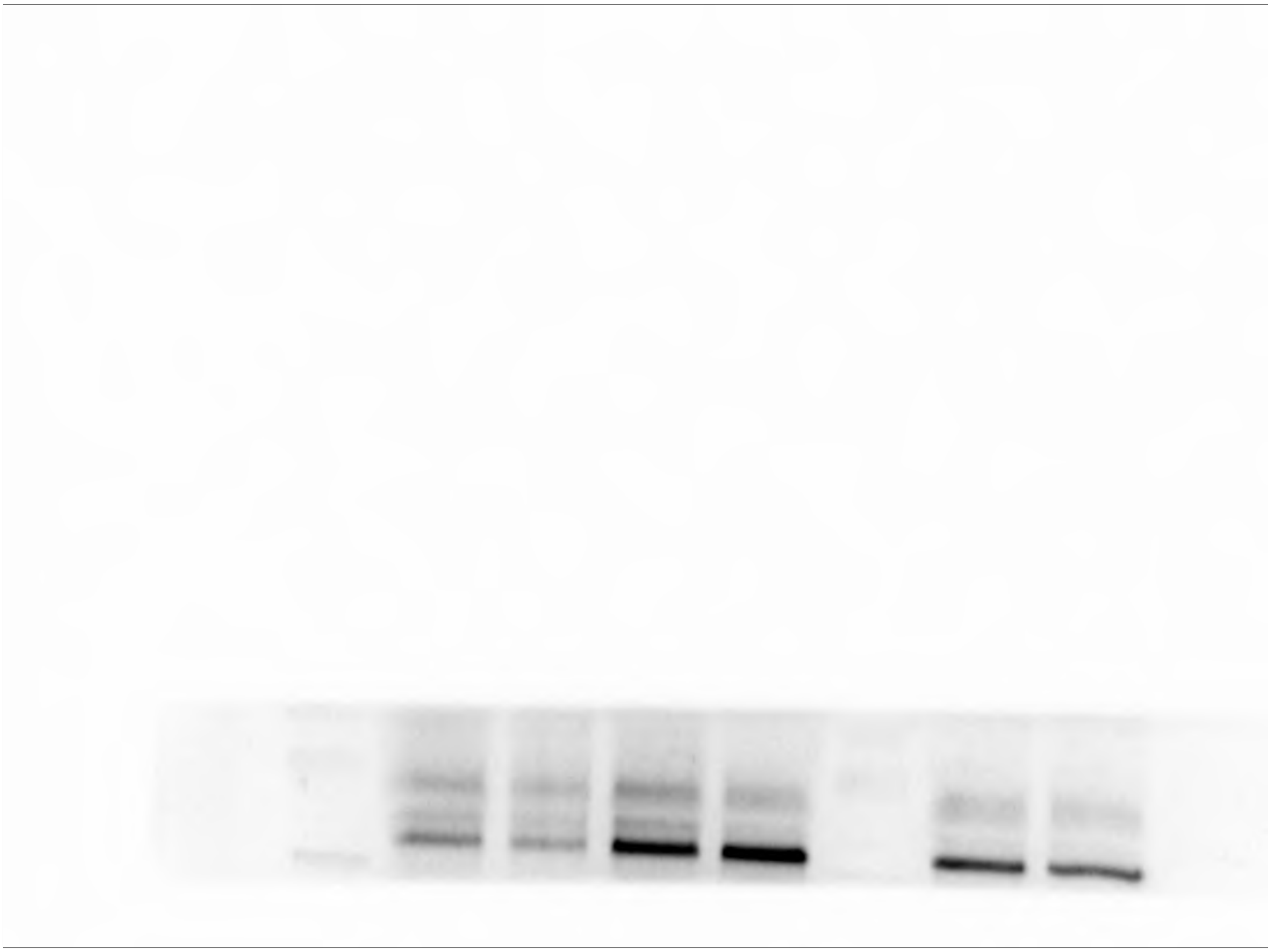

7.vim1.1-sh-NC-K,sh-T-K

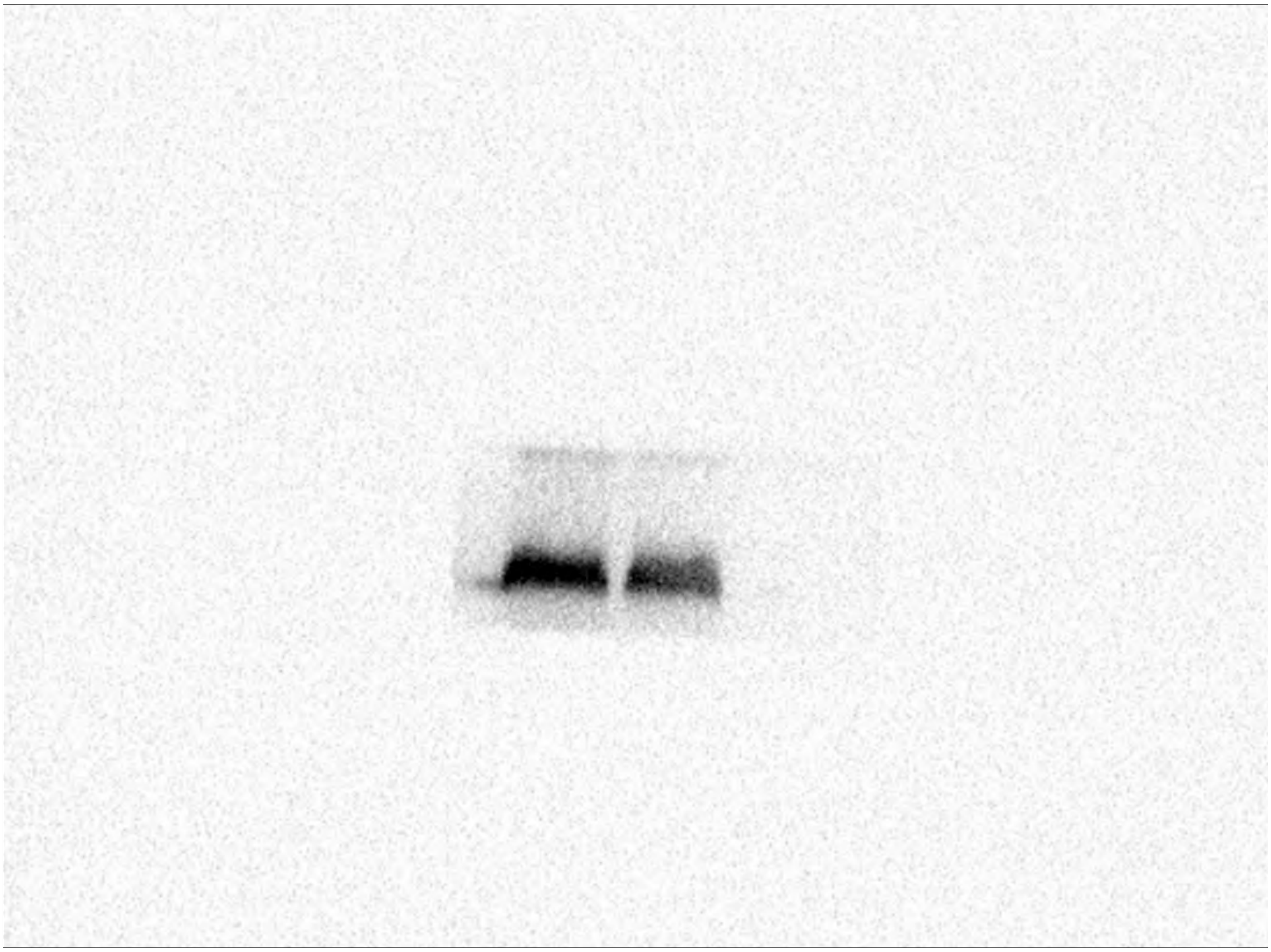

8✔GAPDH11-sh-NC-K,sh-T-K

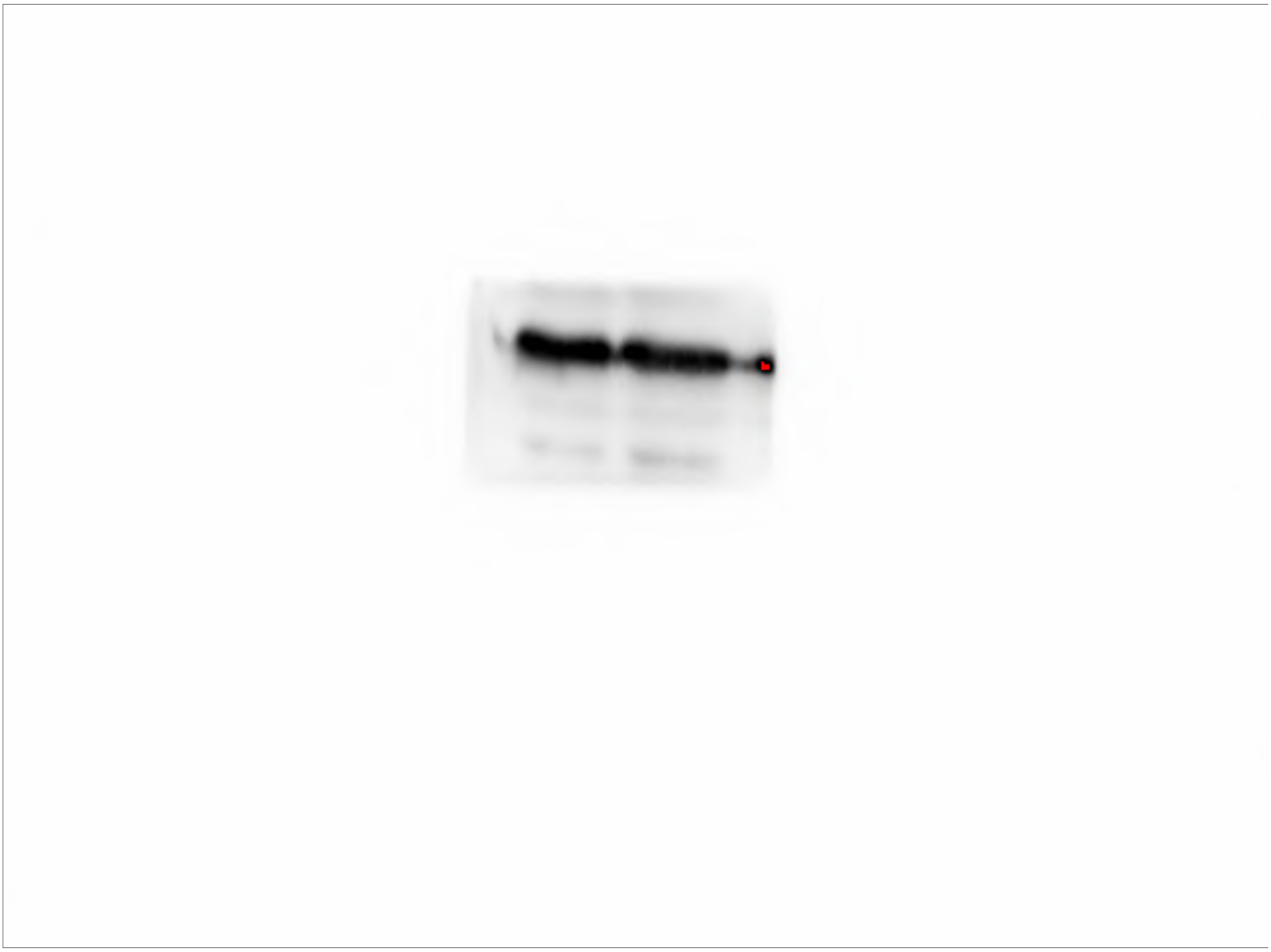

1.Eca-sh-NC-B,sh-T-B,NC mi,16-5p mi,NC in,16-5p in,660,1309,NC

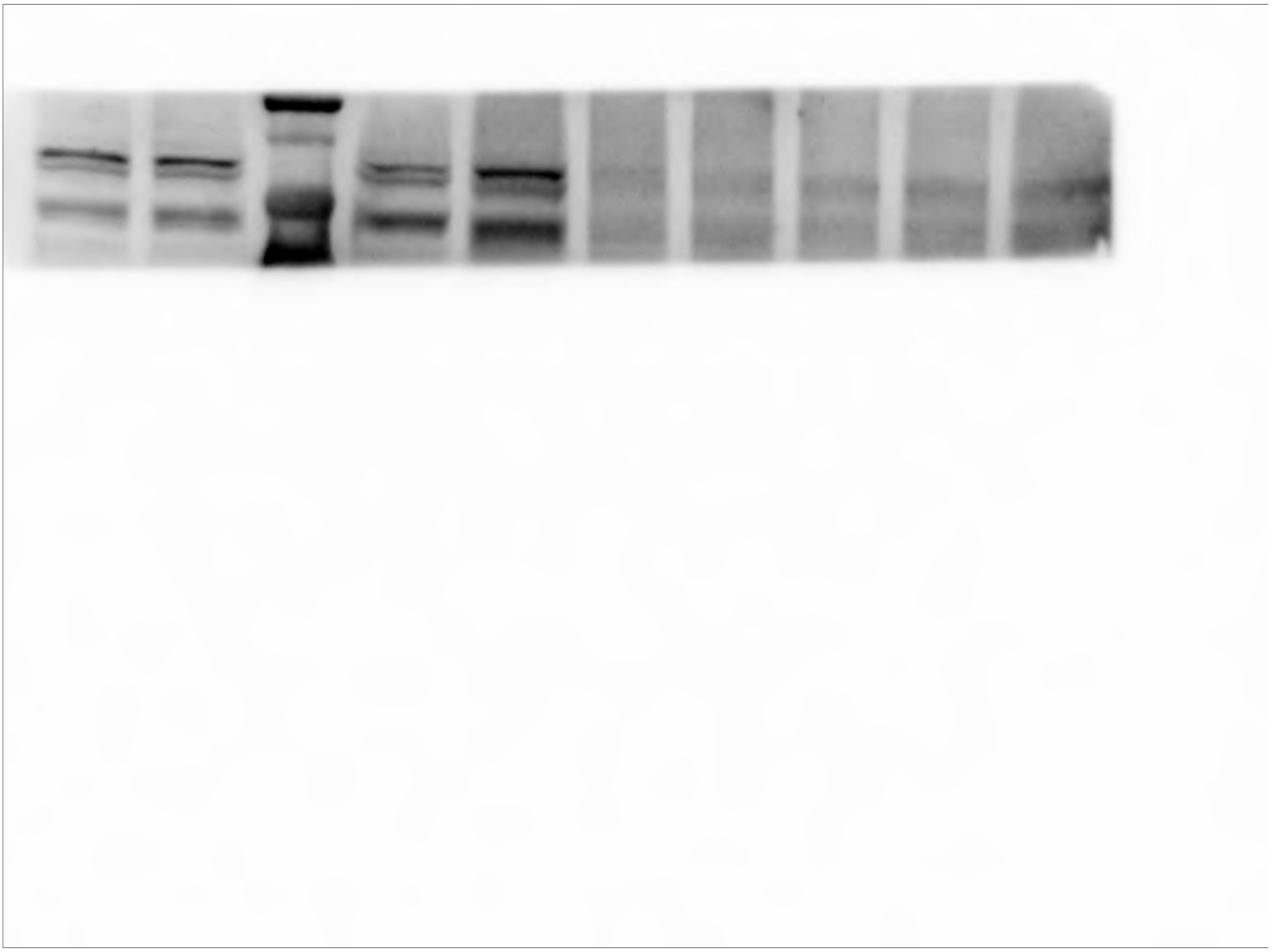

2.Nca1.1-NC mi,16-5p mi,NC mi,16-5p mi

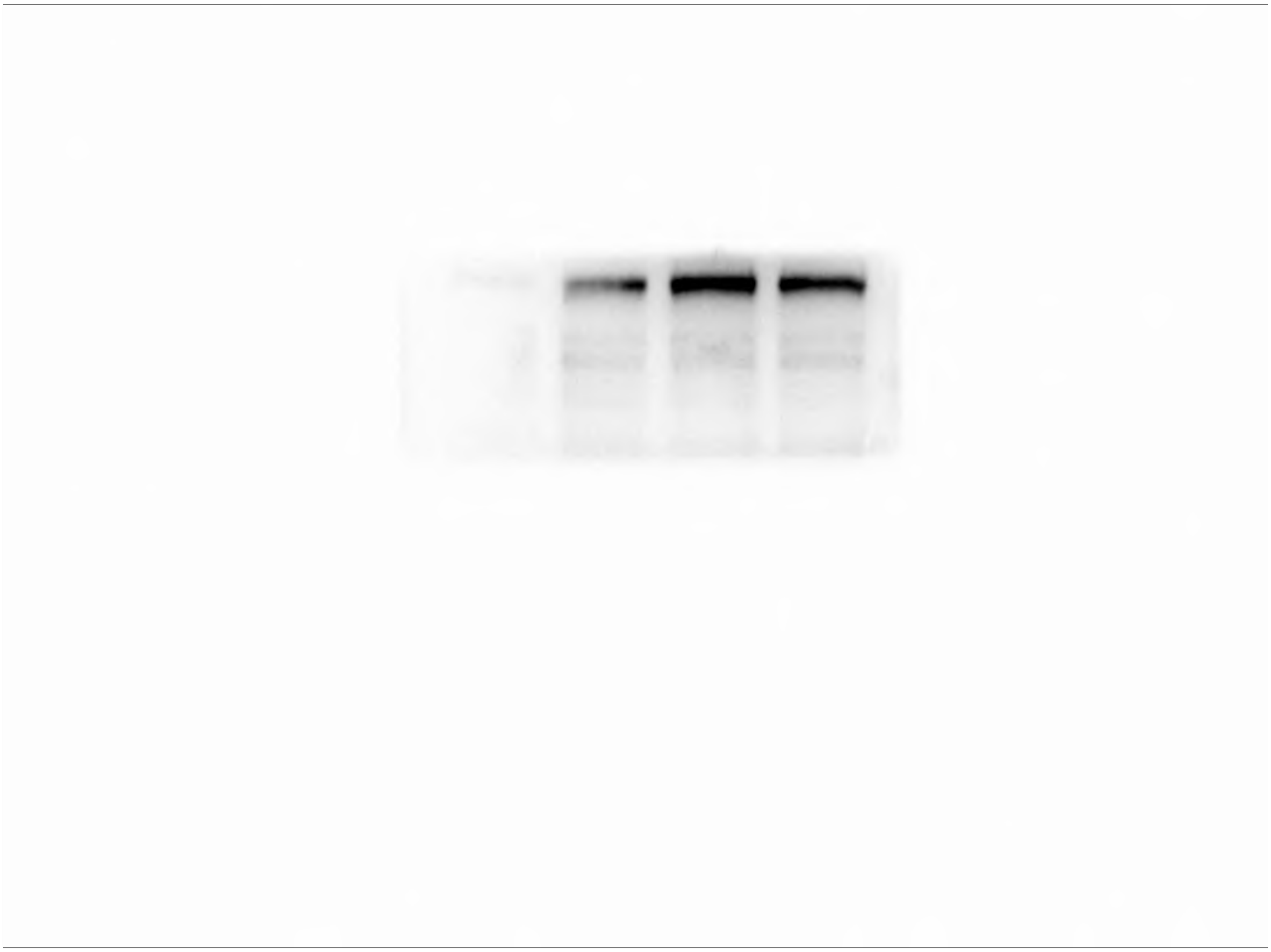

3.vim2.1-NC mi,16-5p mi

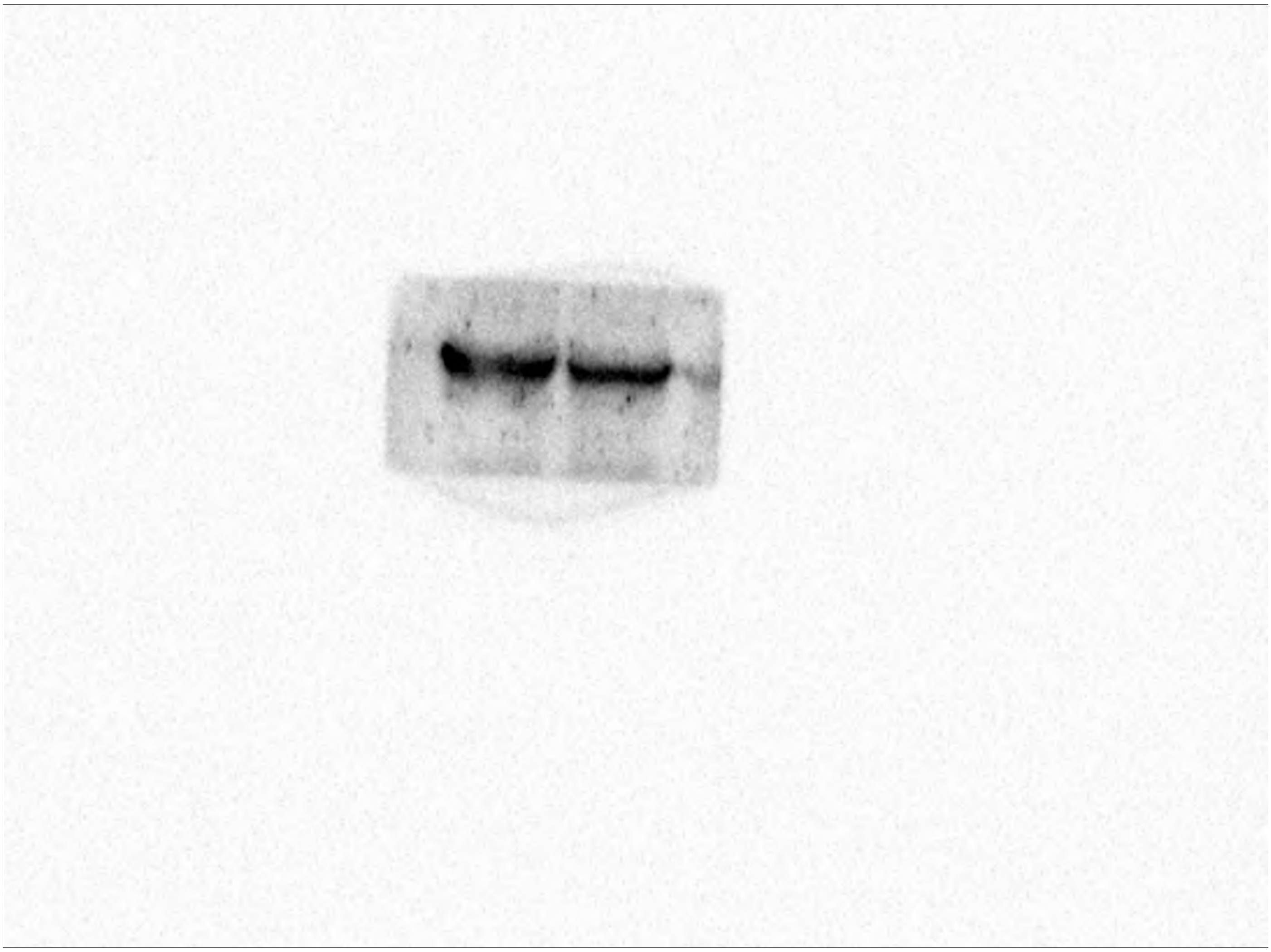

4.GAPDH2.2-NC mi-K,16-5p mi-K

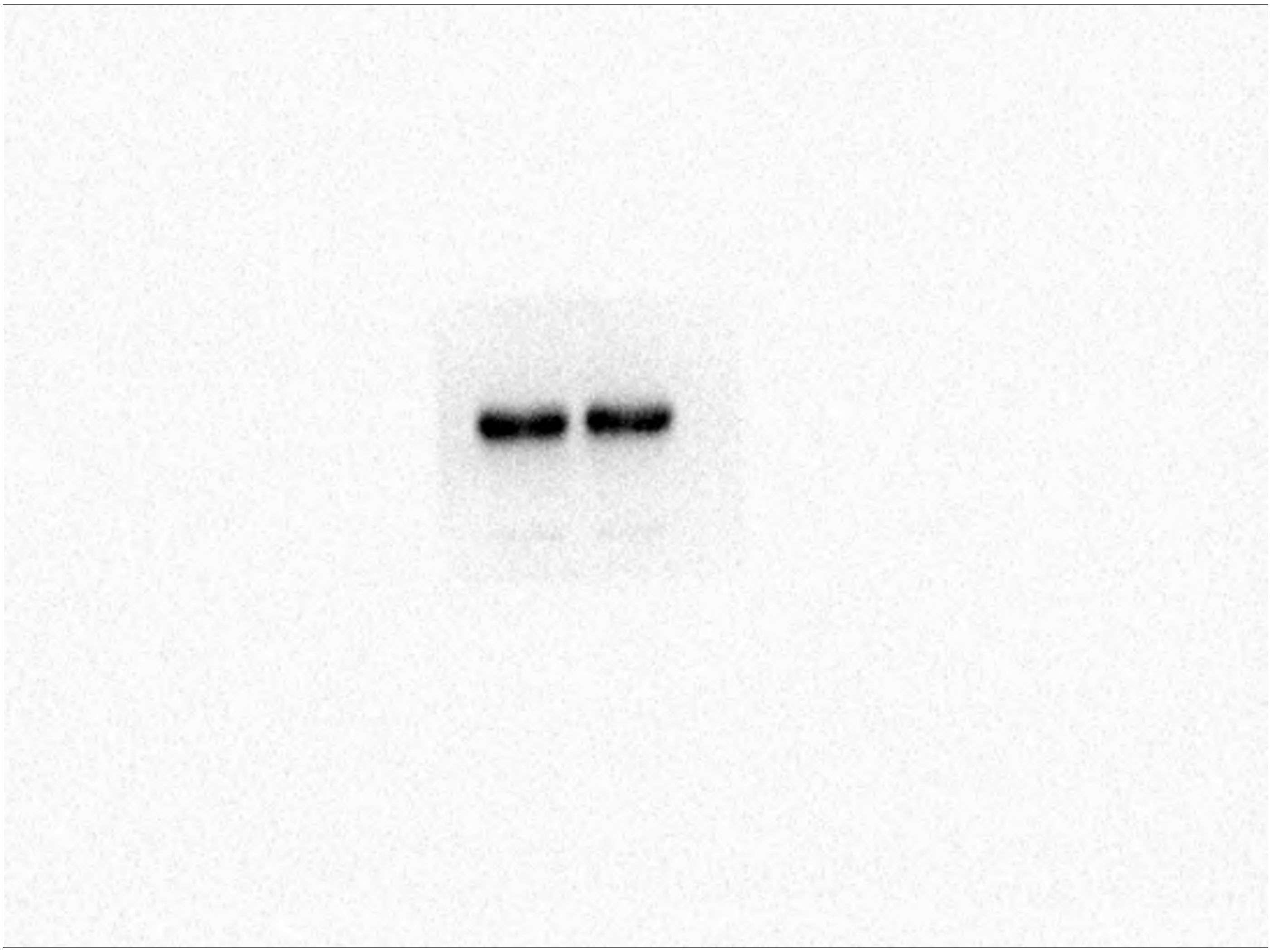

5.✔Eca2-NC mi,16-5p mi,NC in,16-5p in

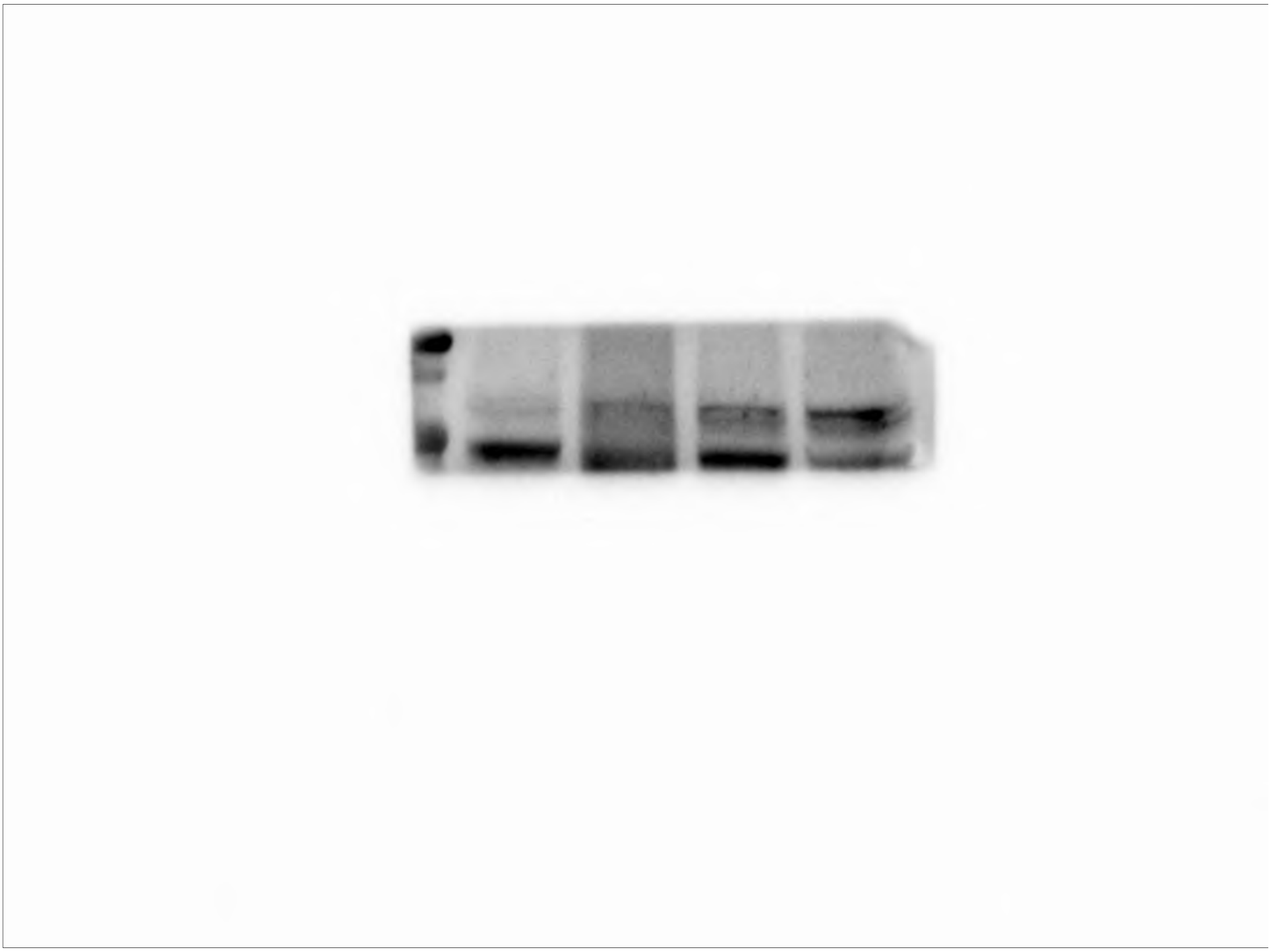

6.Nca-sh-NC,sh-T,NC mi,16-5p mi,NC in,16-5p in

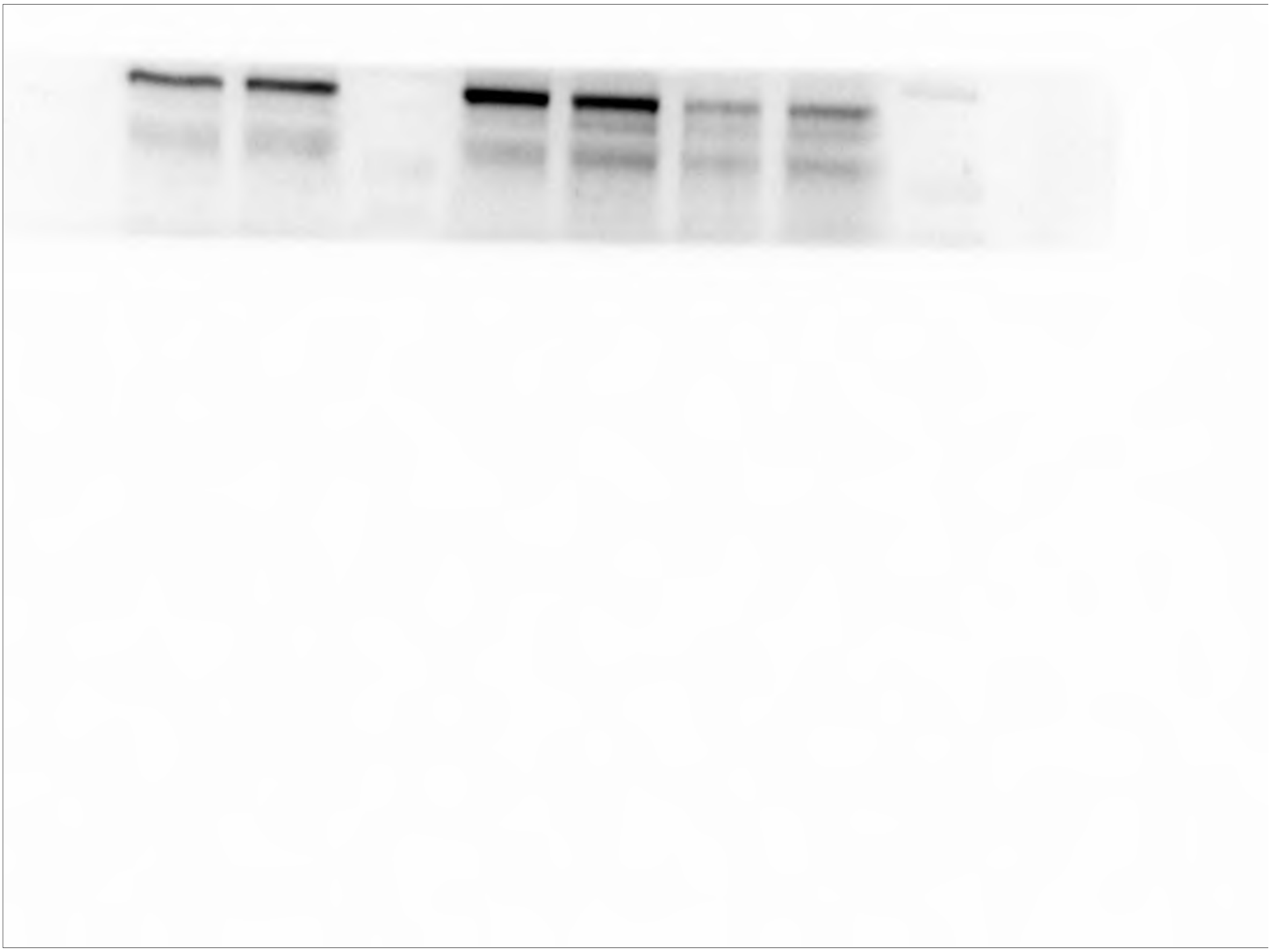

7.vim6.2-NC mi,16-5p mi

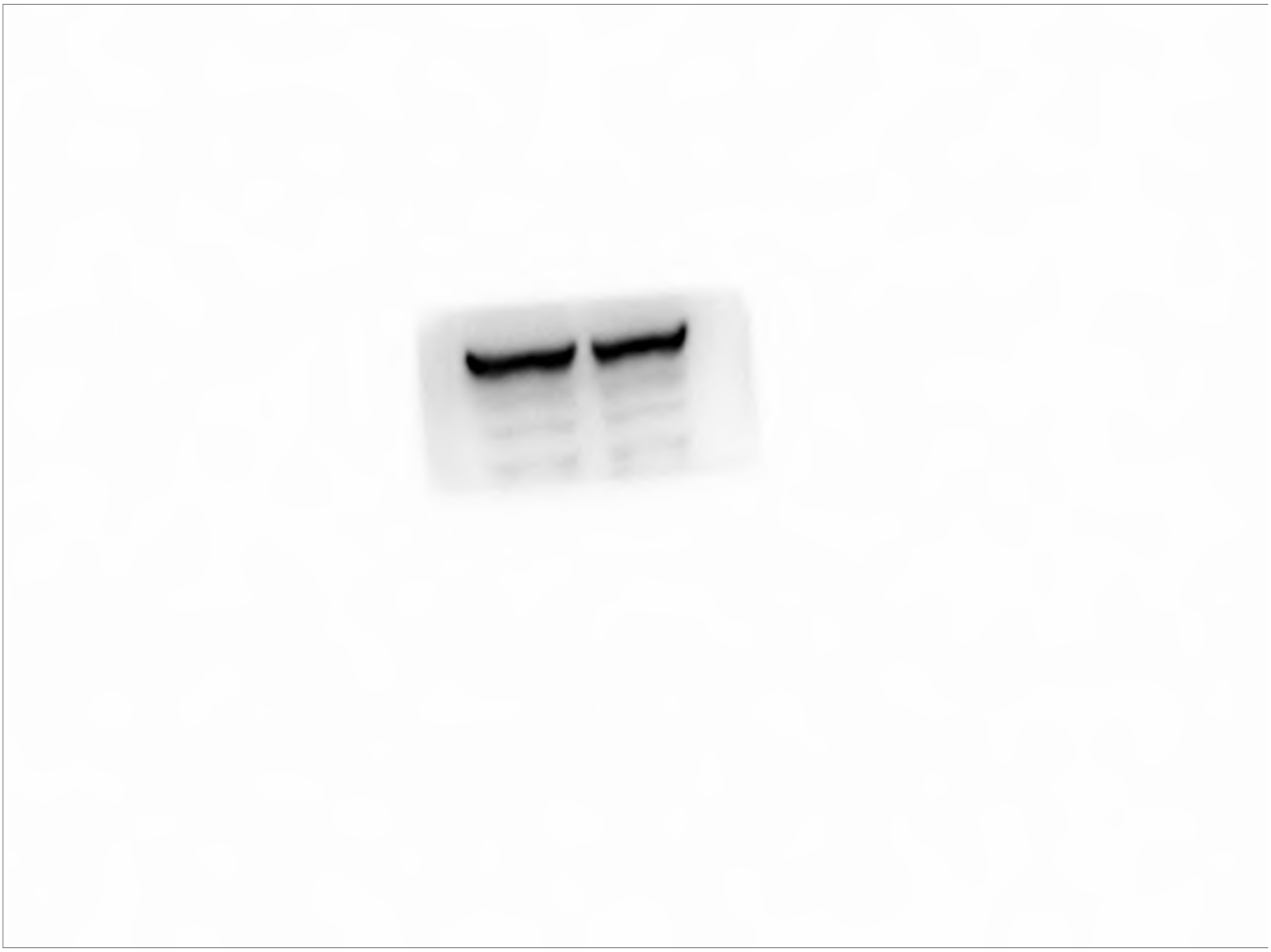

8.GAPDH5.2-NC mi-K,16-5p mi-K

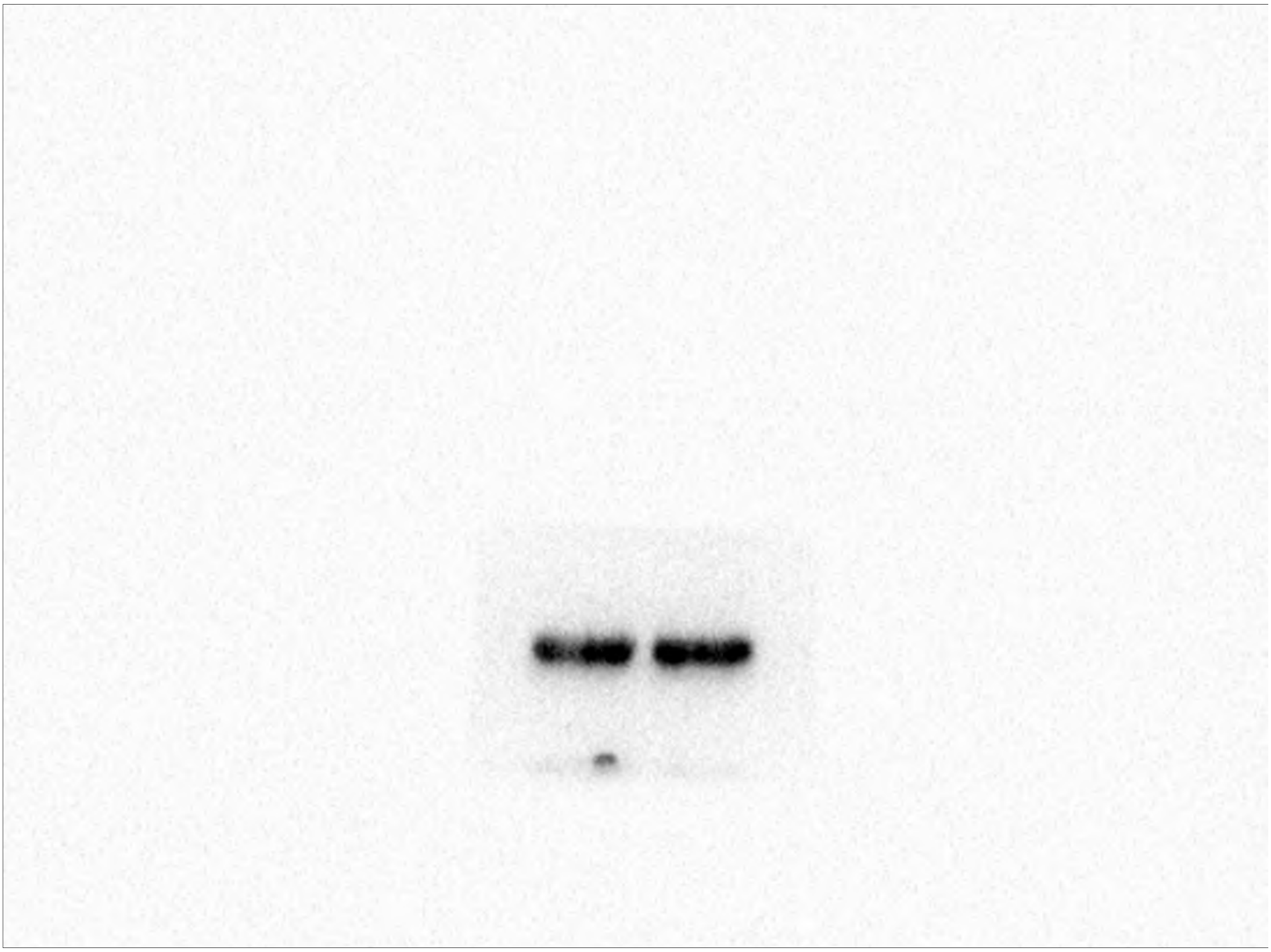

1.Eca1.1-NC in,16-5p in

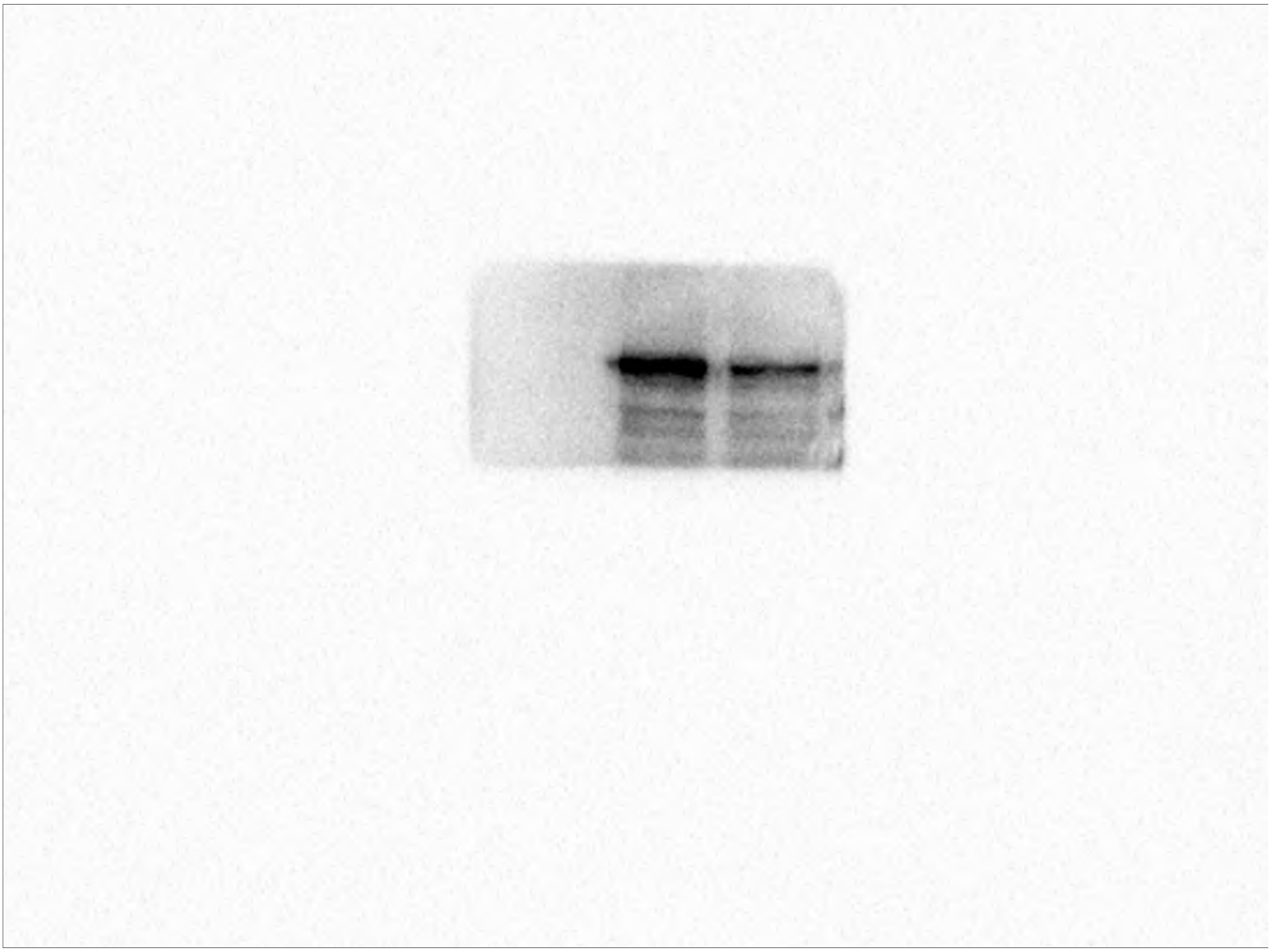

2.Nca2.1-NC in,16-5p in

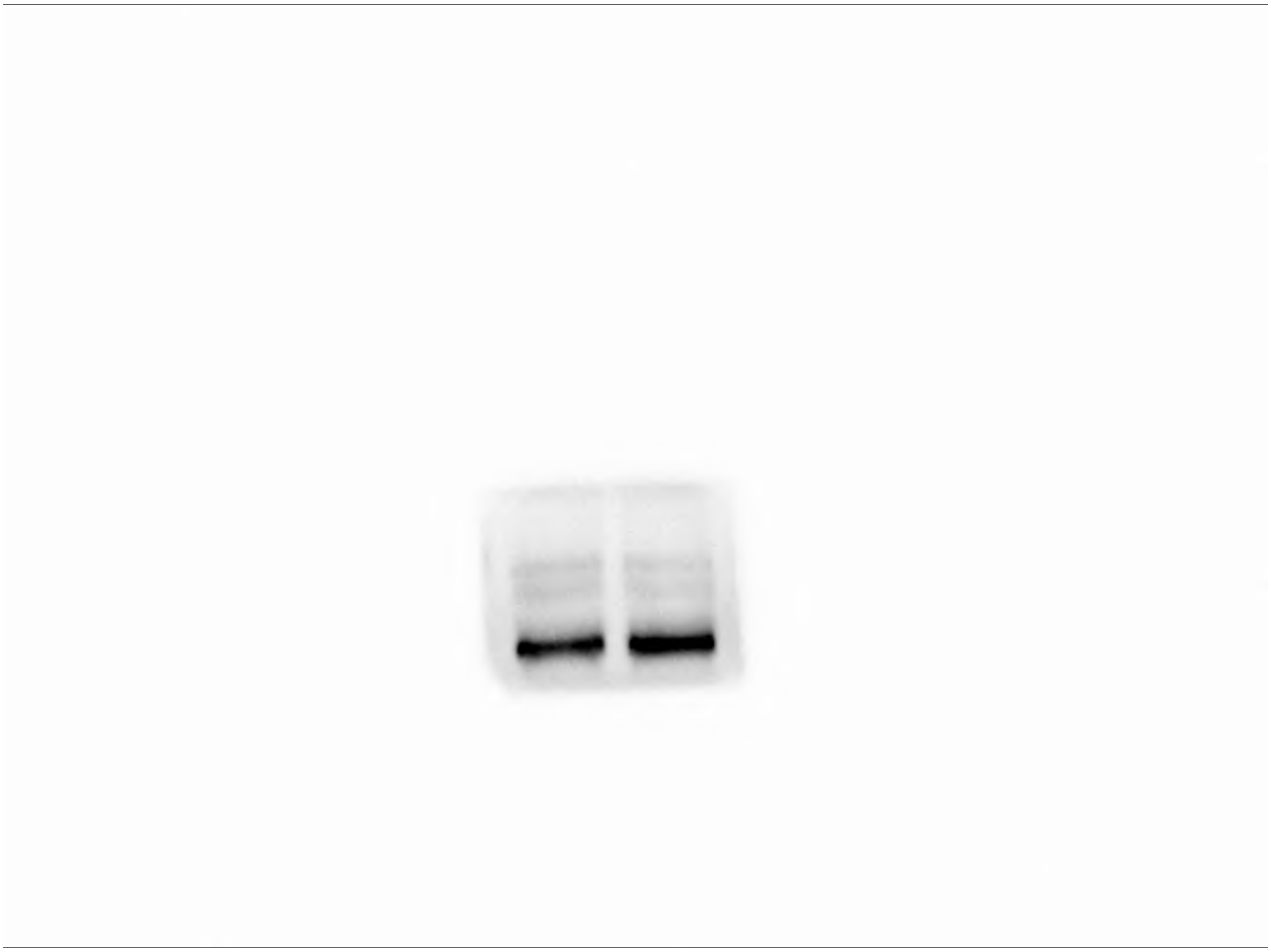

3.vim3.1-NC in-B,16-5p in-B

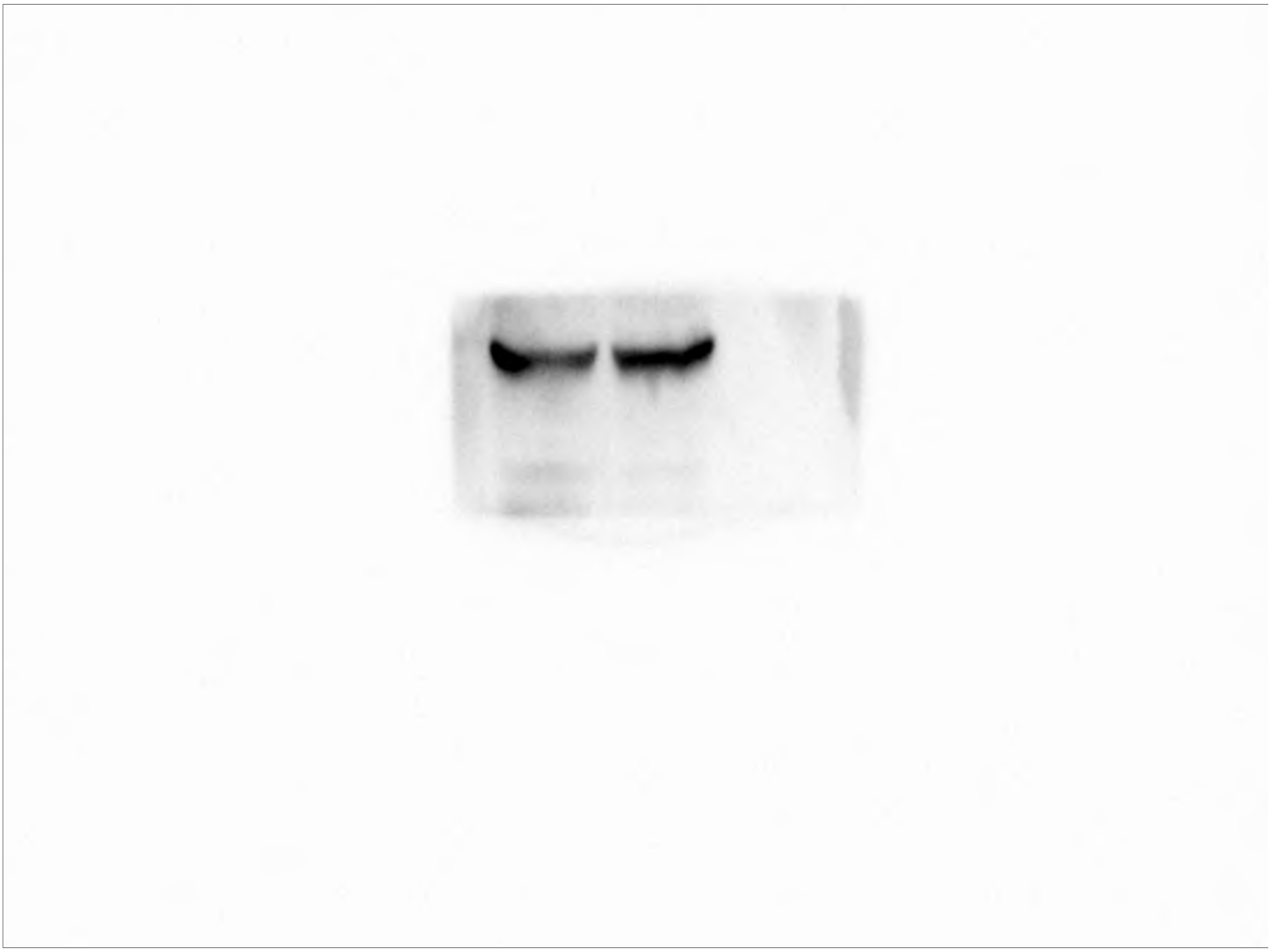

4.GAPDH2.3-NC in,16-5p in

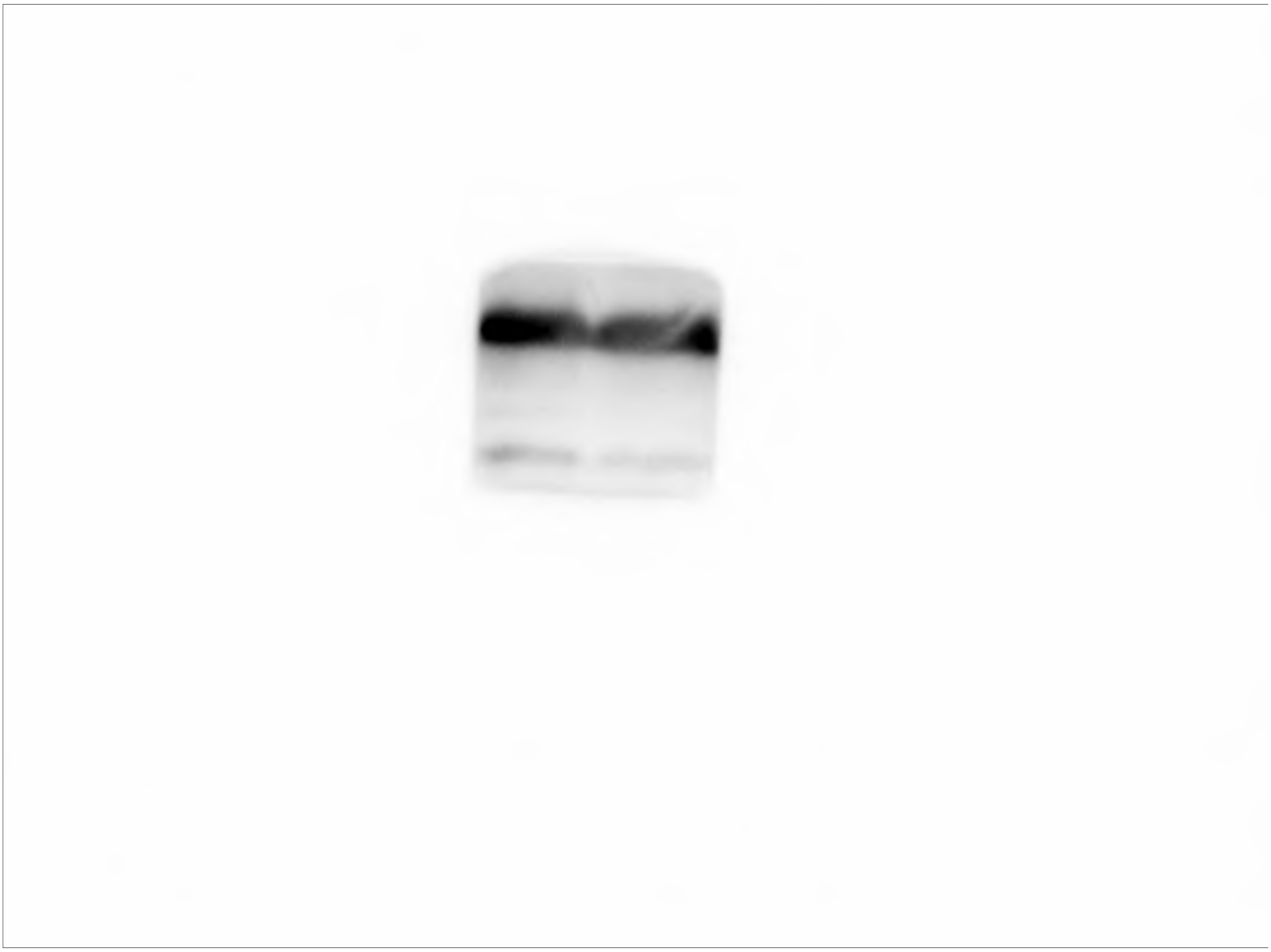

5.Eca4.1.1-NC in,16-5p in

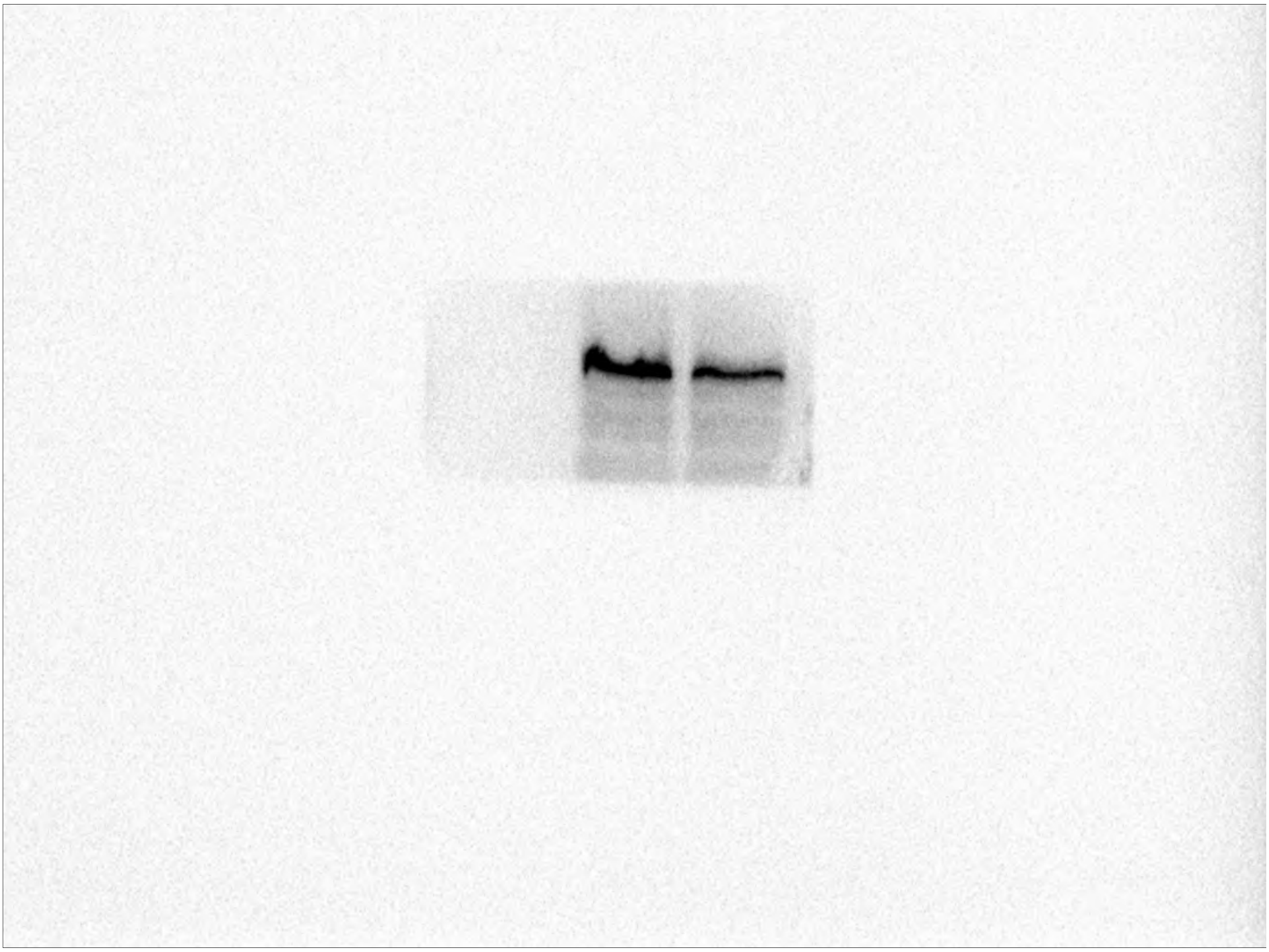

6.Nca6.1.4-NC in,16-5p in

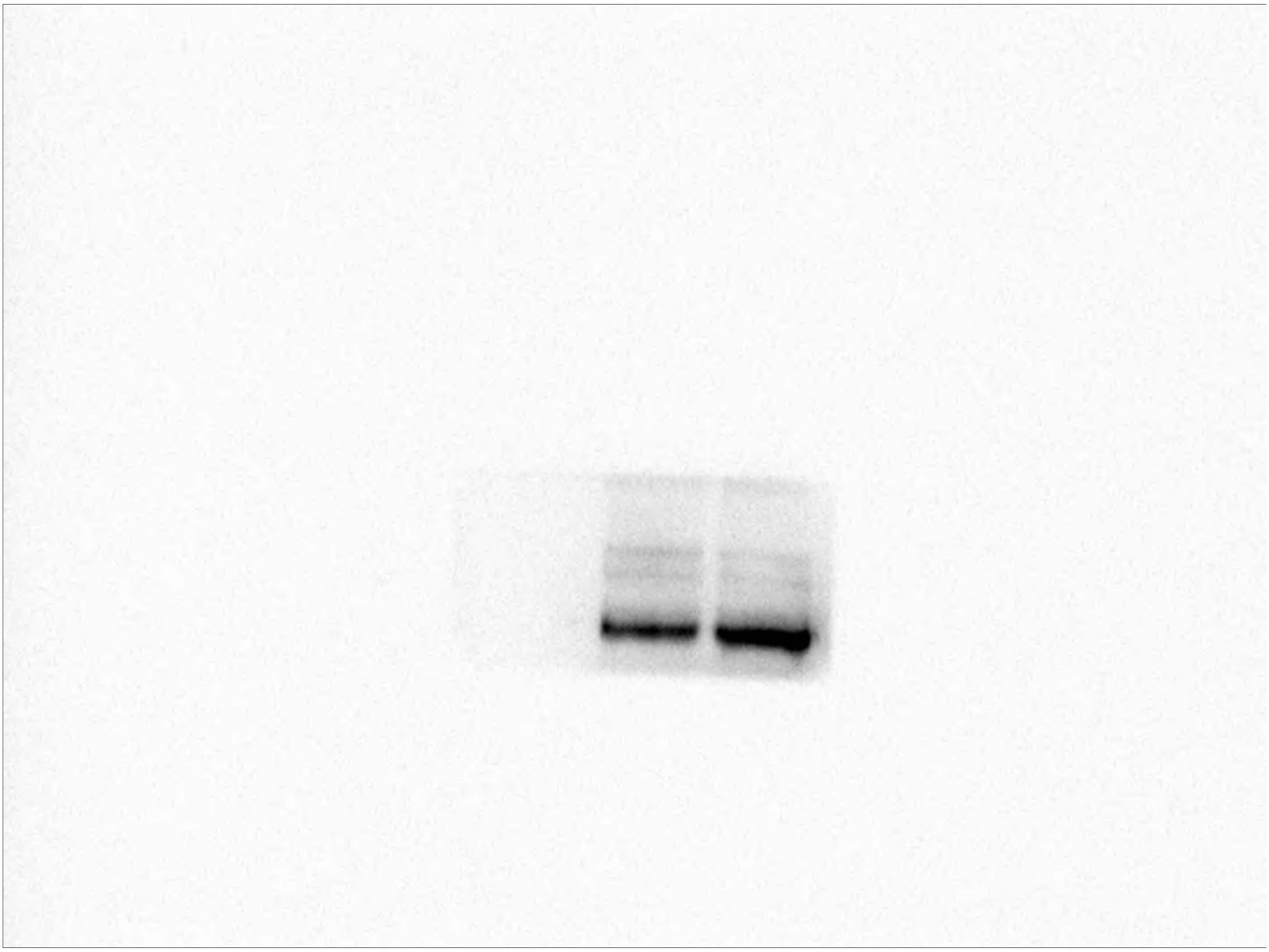

7.vim3.1-NC in K,16-5p in K

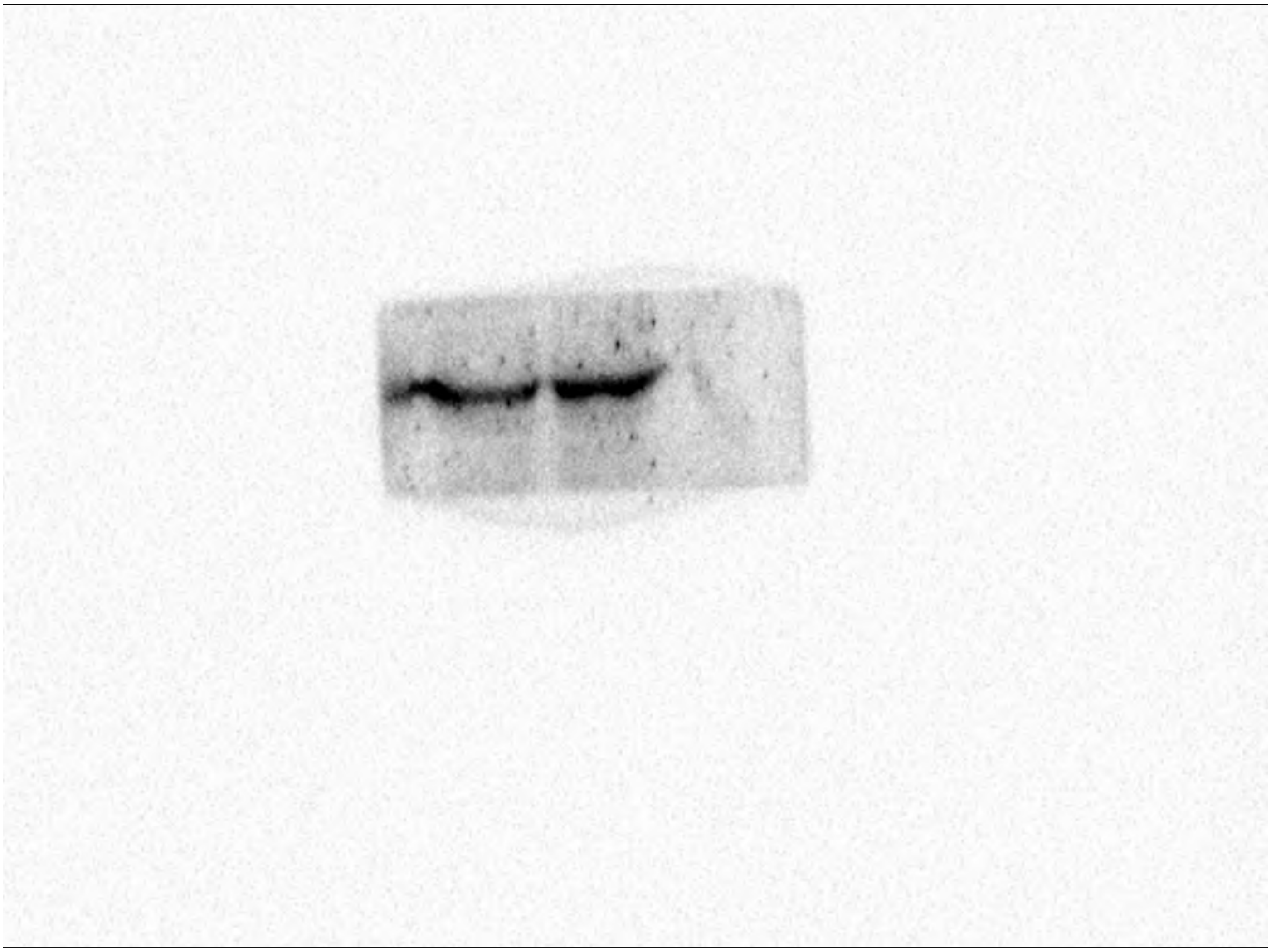

8.GAPDH6.3-NC in,16-5p in

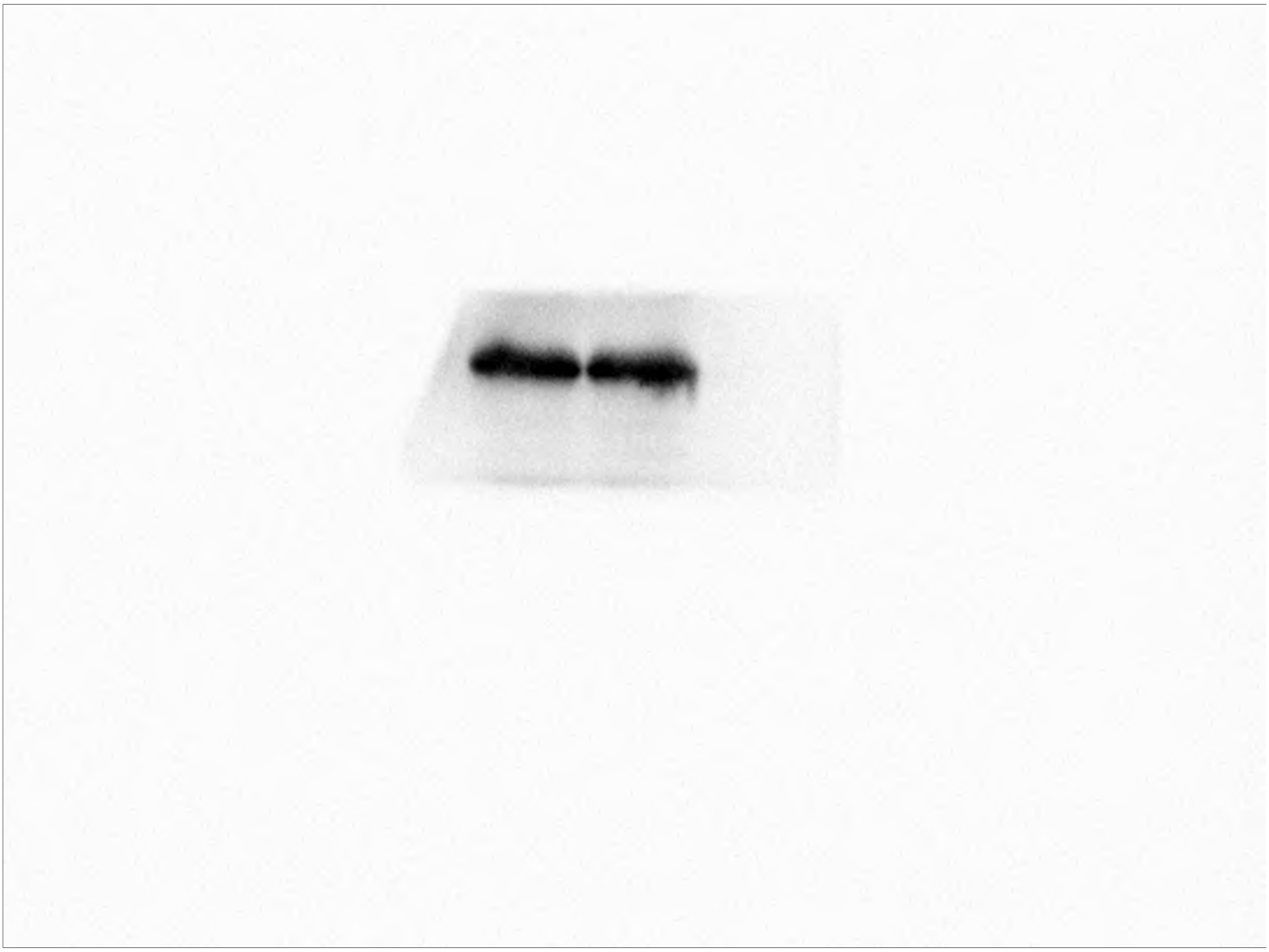

9.Smad3 4.2-NC in-B,16-5p in-B

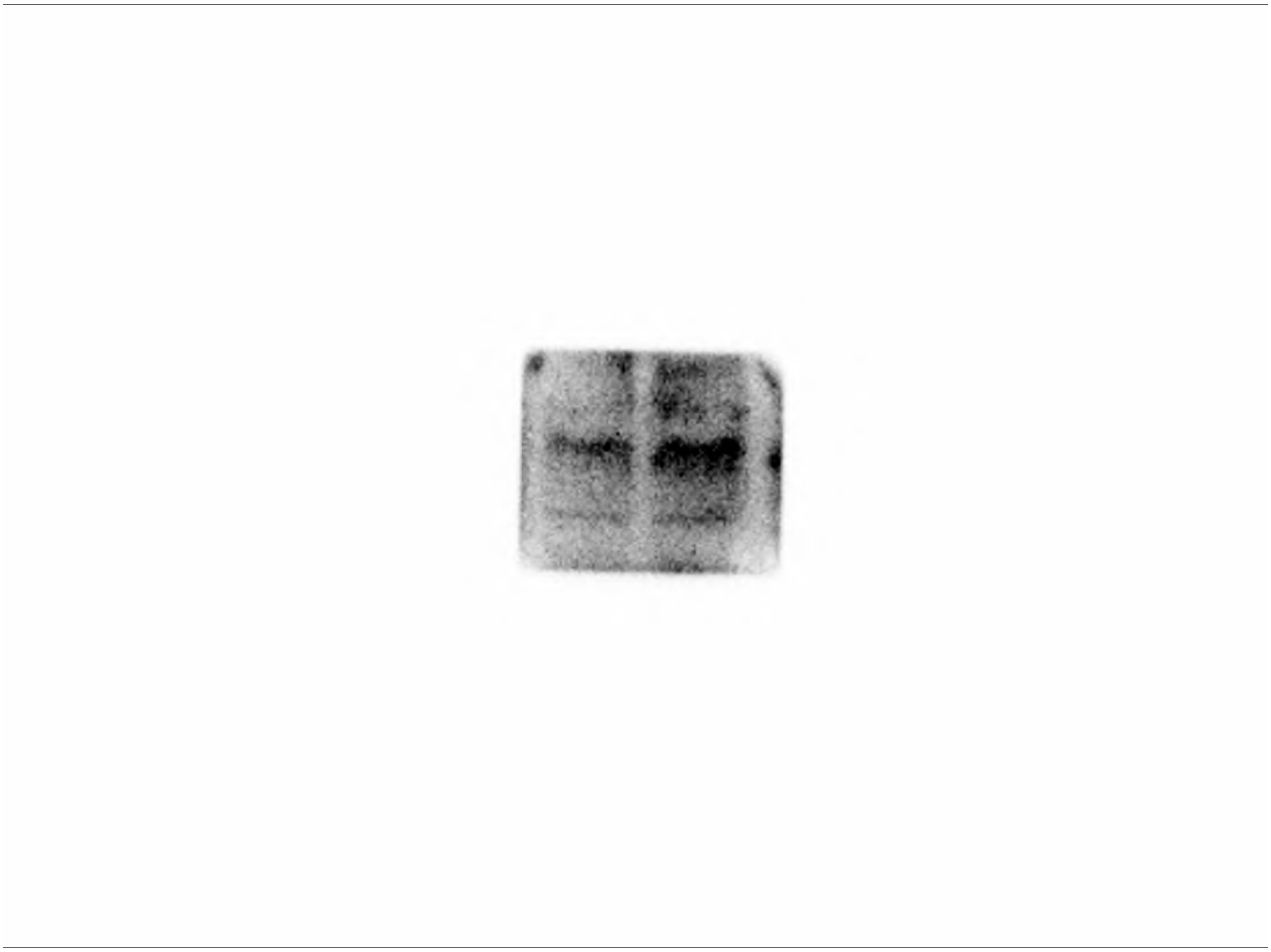

10.GAPDH3.2-NC in-B,16-5p in-B

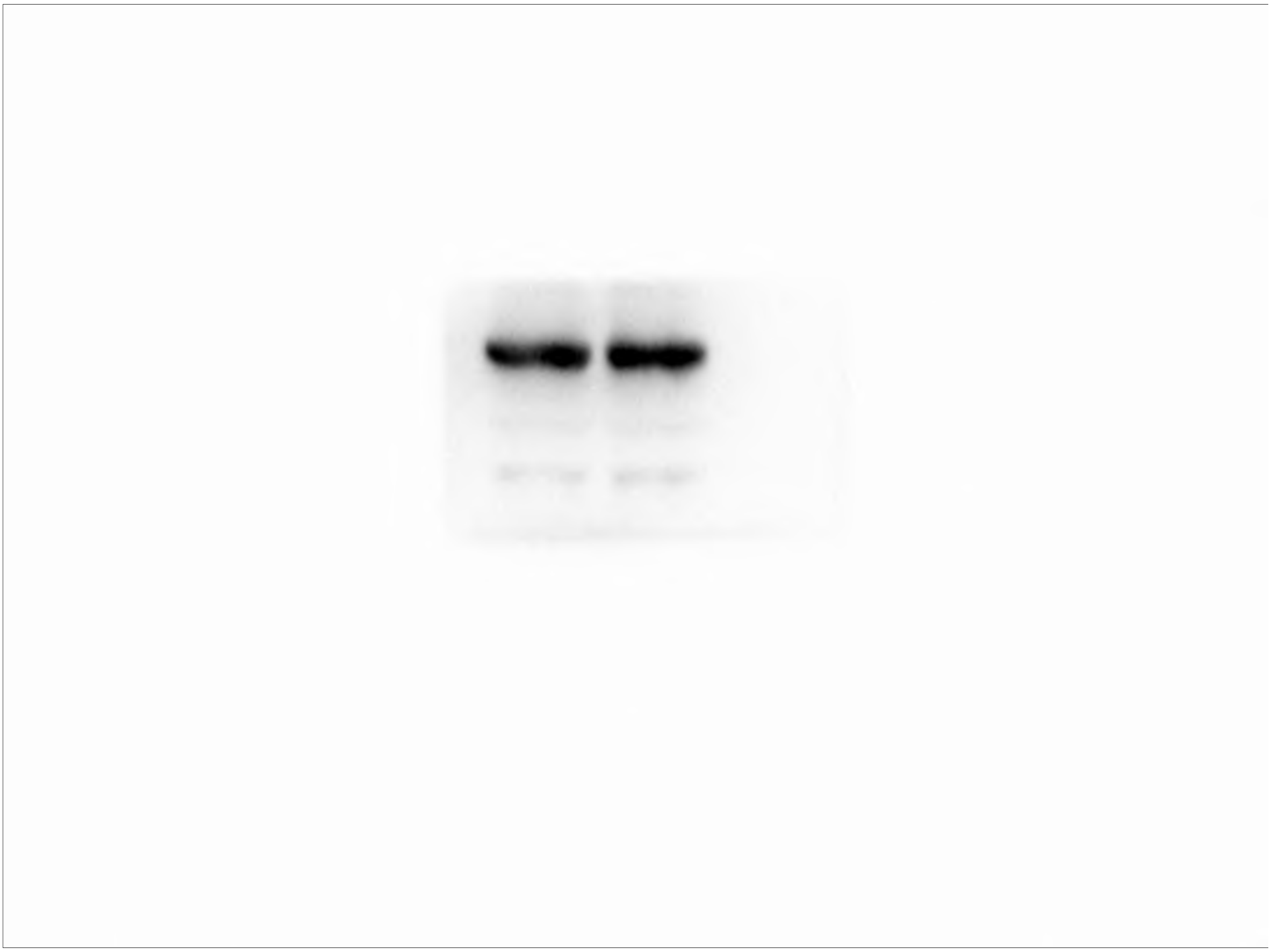

11.Smad3 2.2.1-NC in,16-5p in

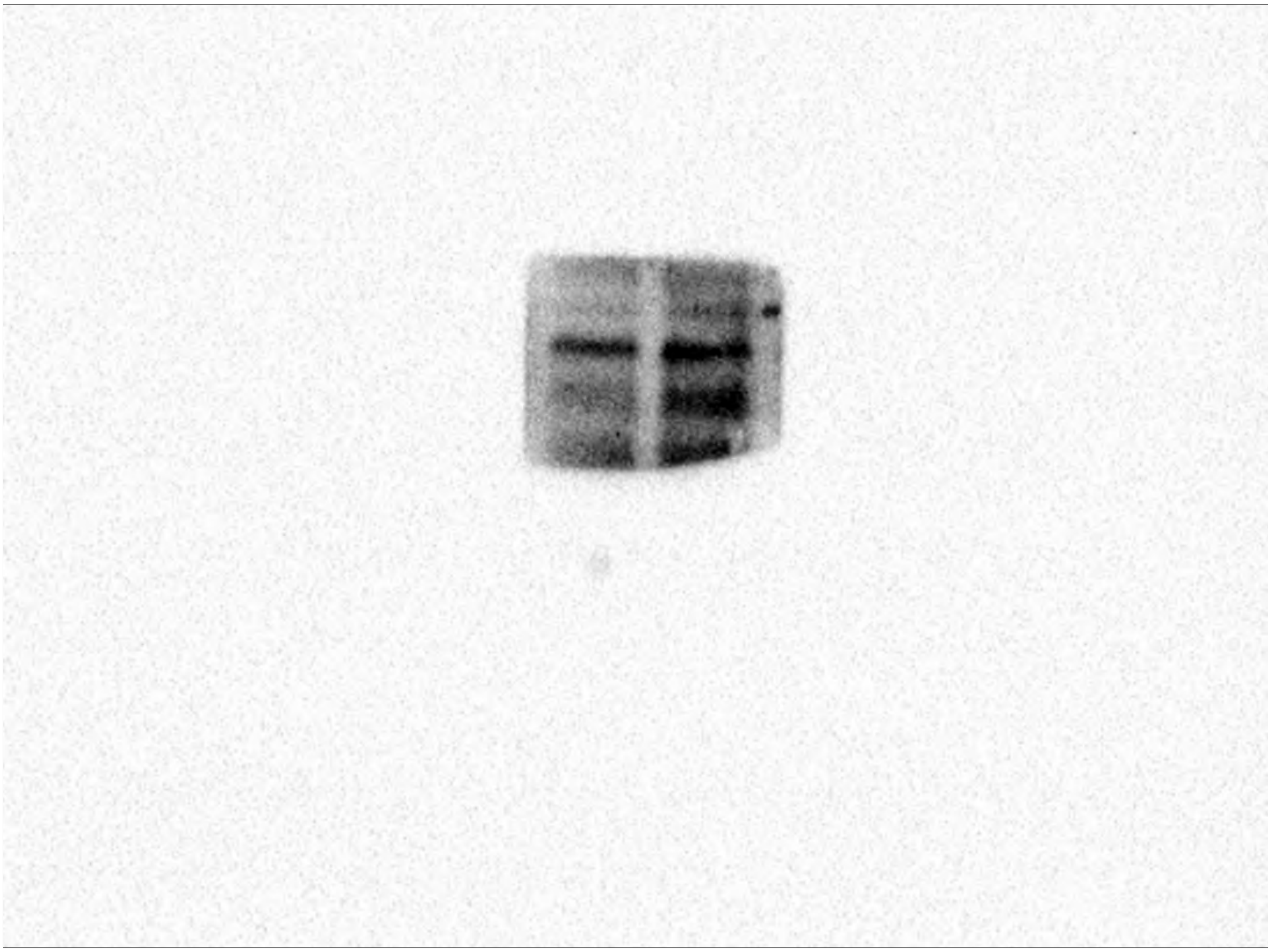

12.GAPDH4.3-NC in-K,16-5p in-K

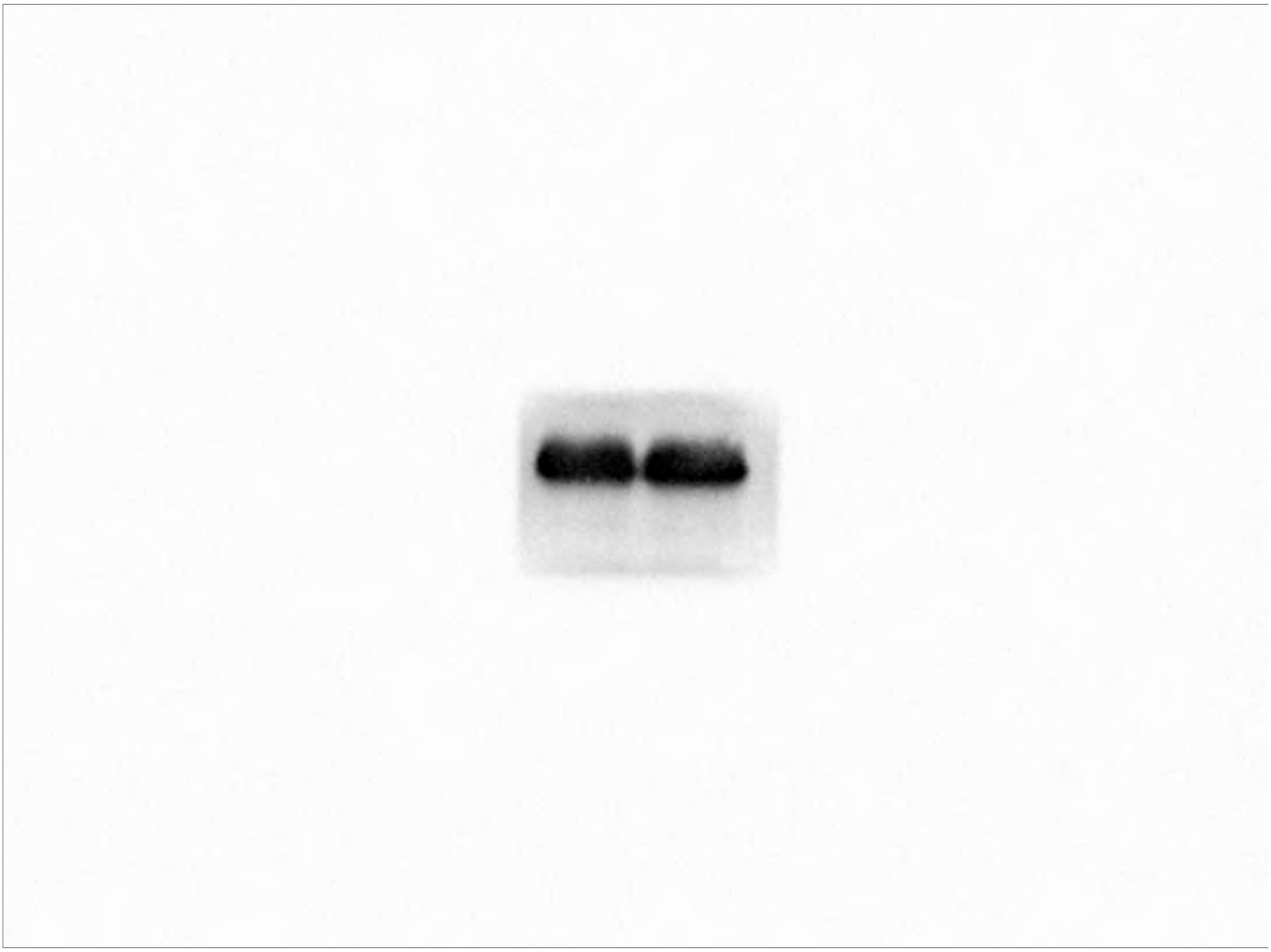

9.Smad3 2.2-NC mi,16-p mi

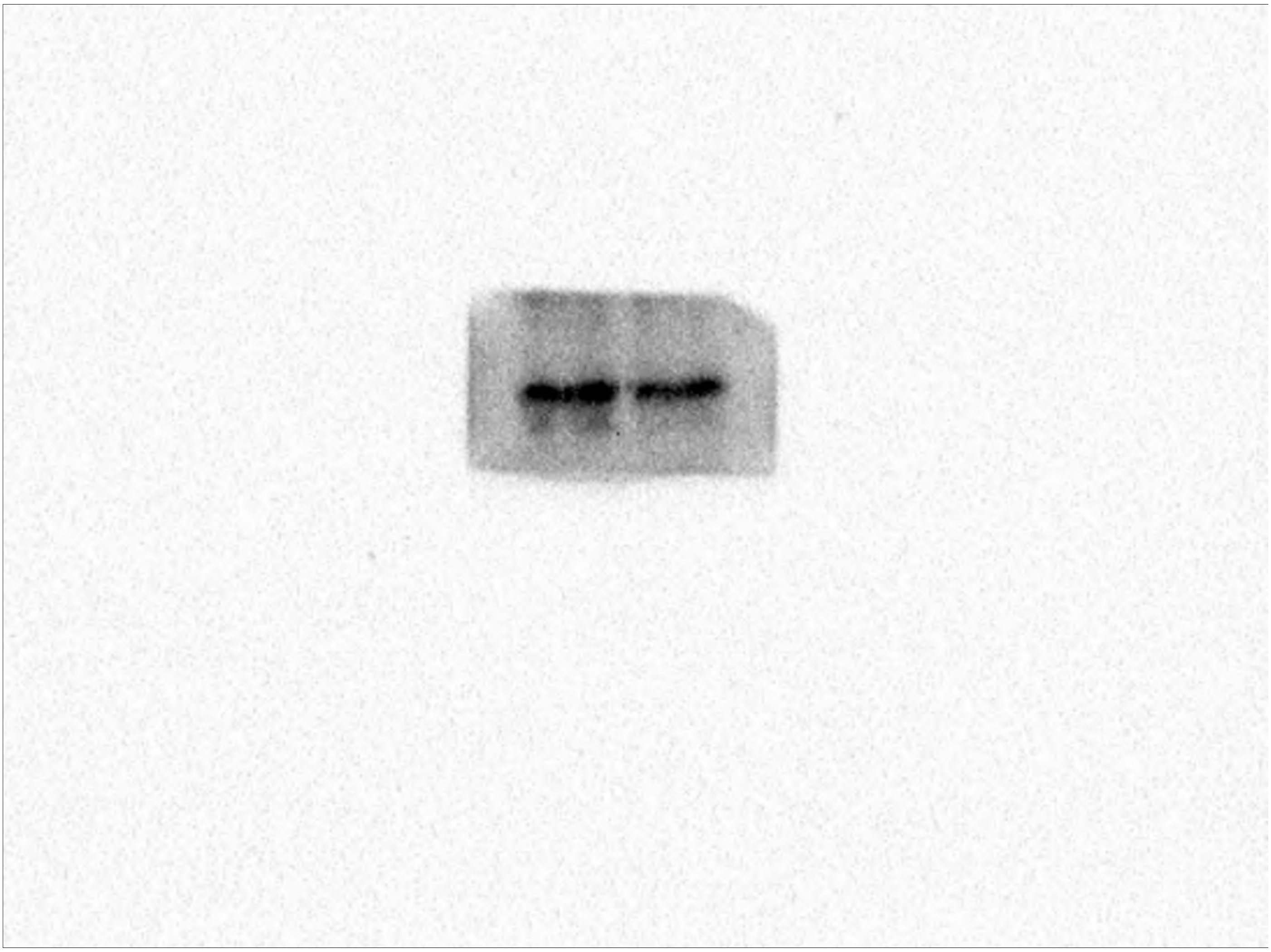

10.GAPDH2.3-NC mi,16-5p mi

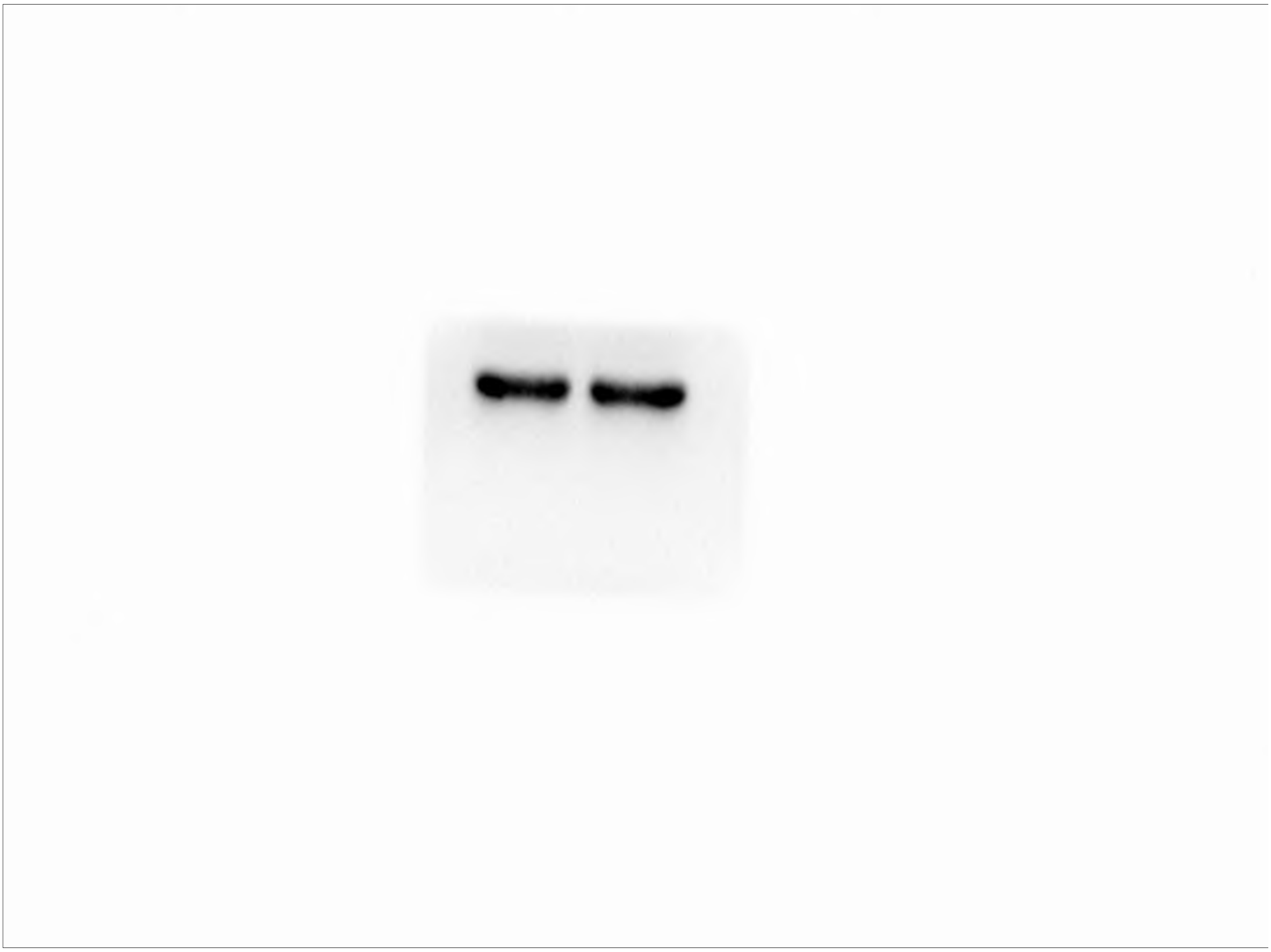

11.Smad3 2.2.2-NC mi,16-5p mi

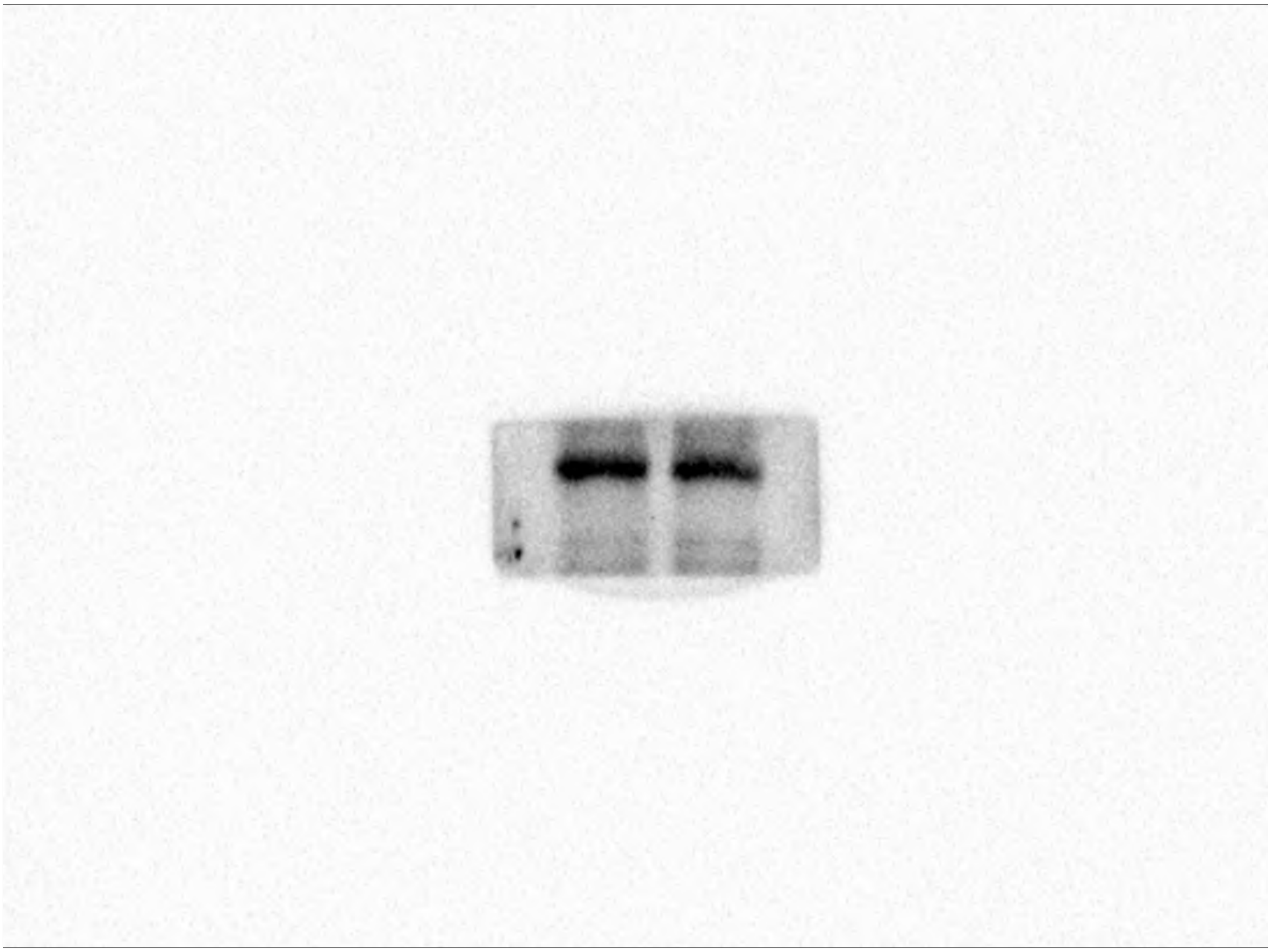

12.GAPDH5.2-NC mi-K,16-5p mi-K

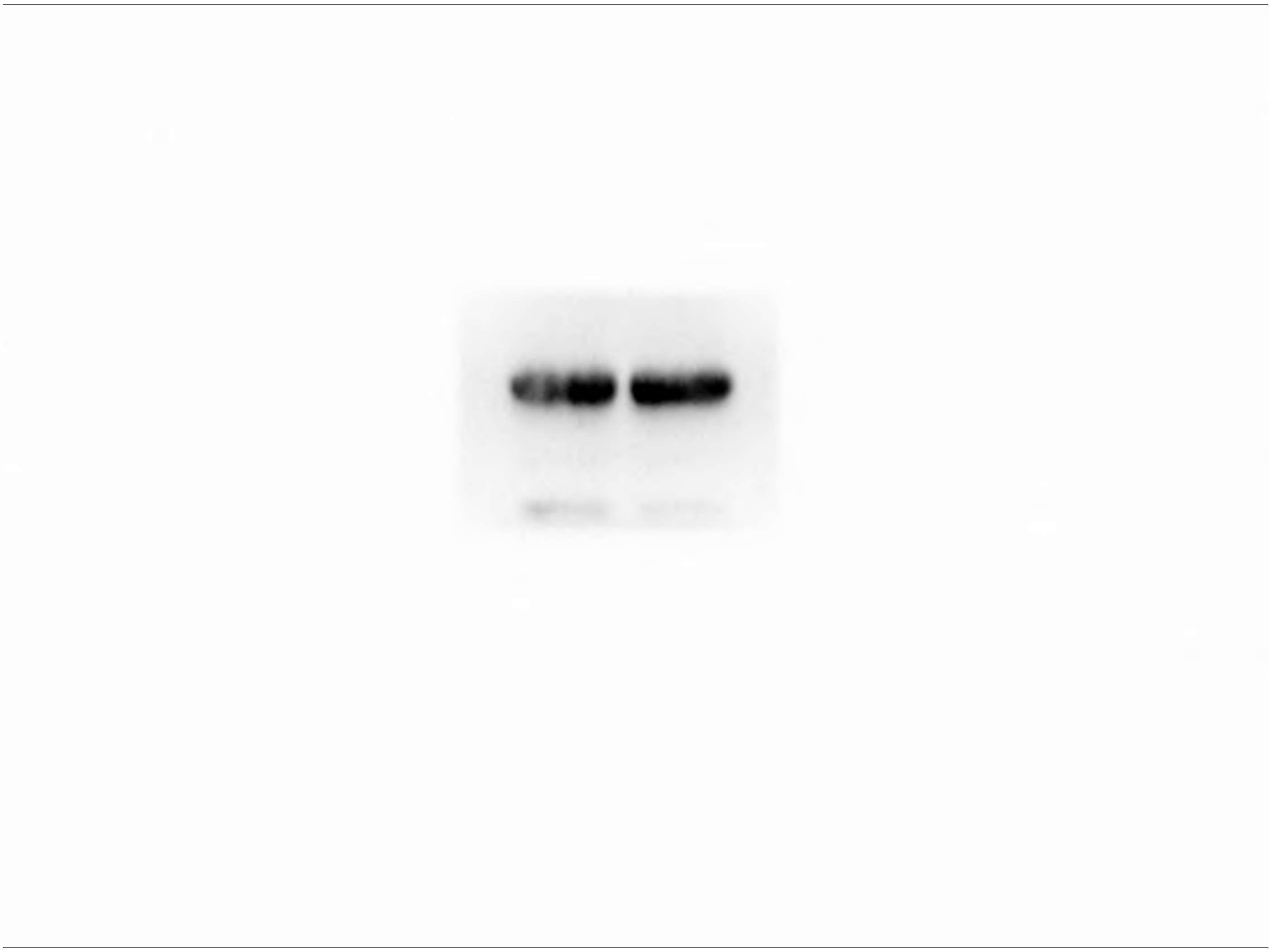

1.✔Smad3 1.2.1-sh-NC-B,sh-T-B

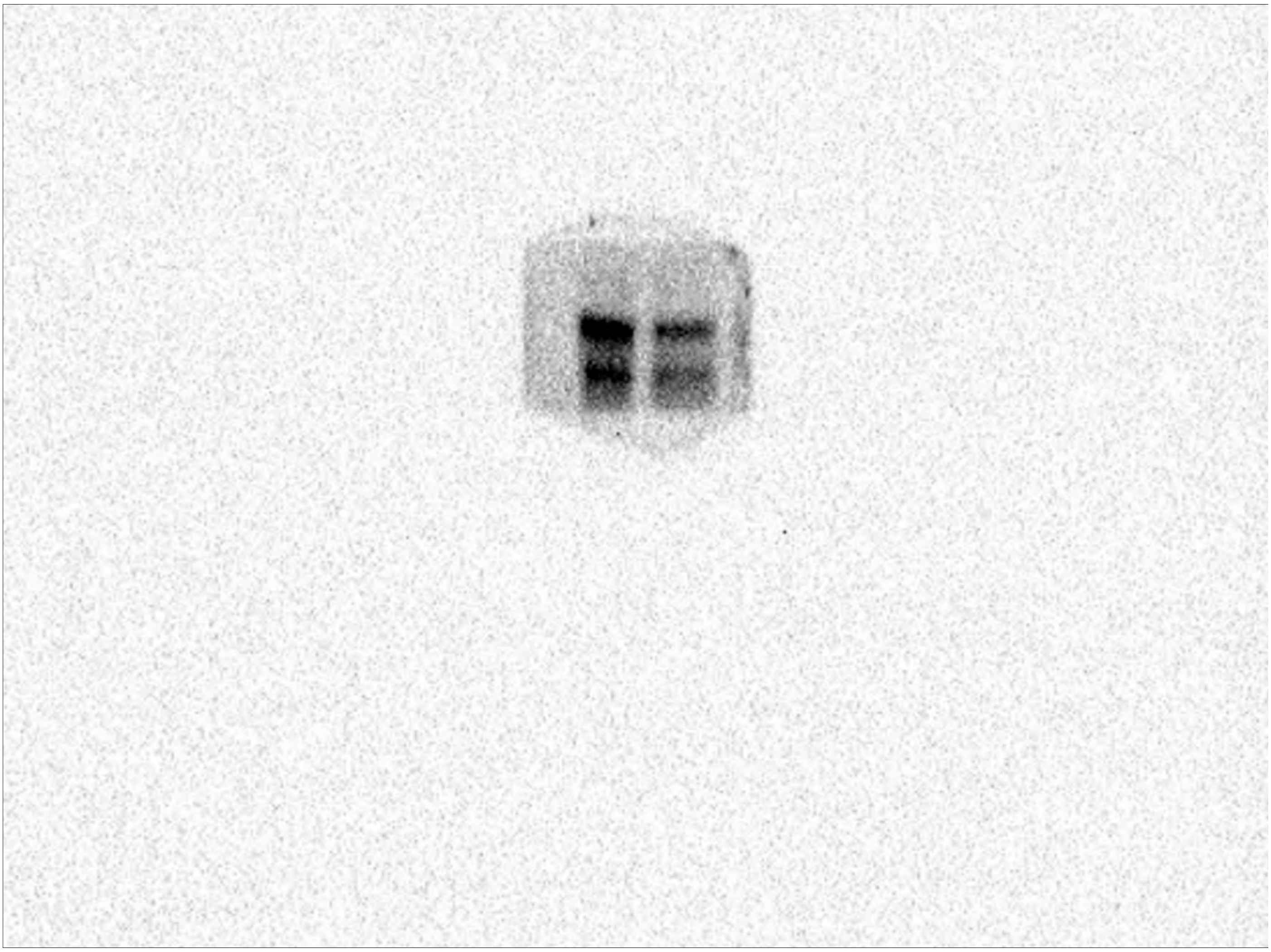

2.GAPDH1.2-sh-NC-B,sh-T-B

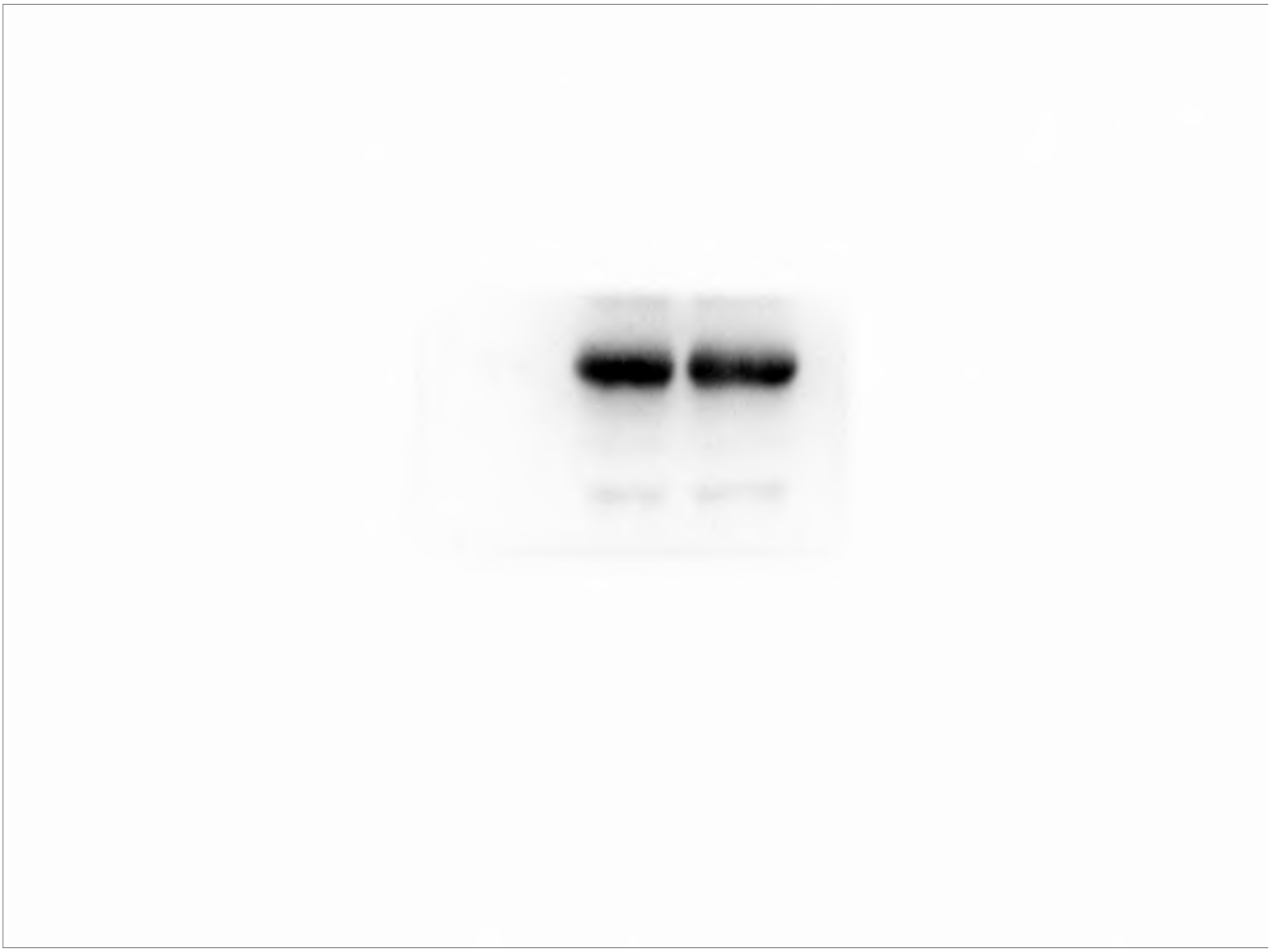

3.✔Smad3 1.2.1-sh-NC-K,sh-T-K

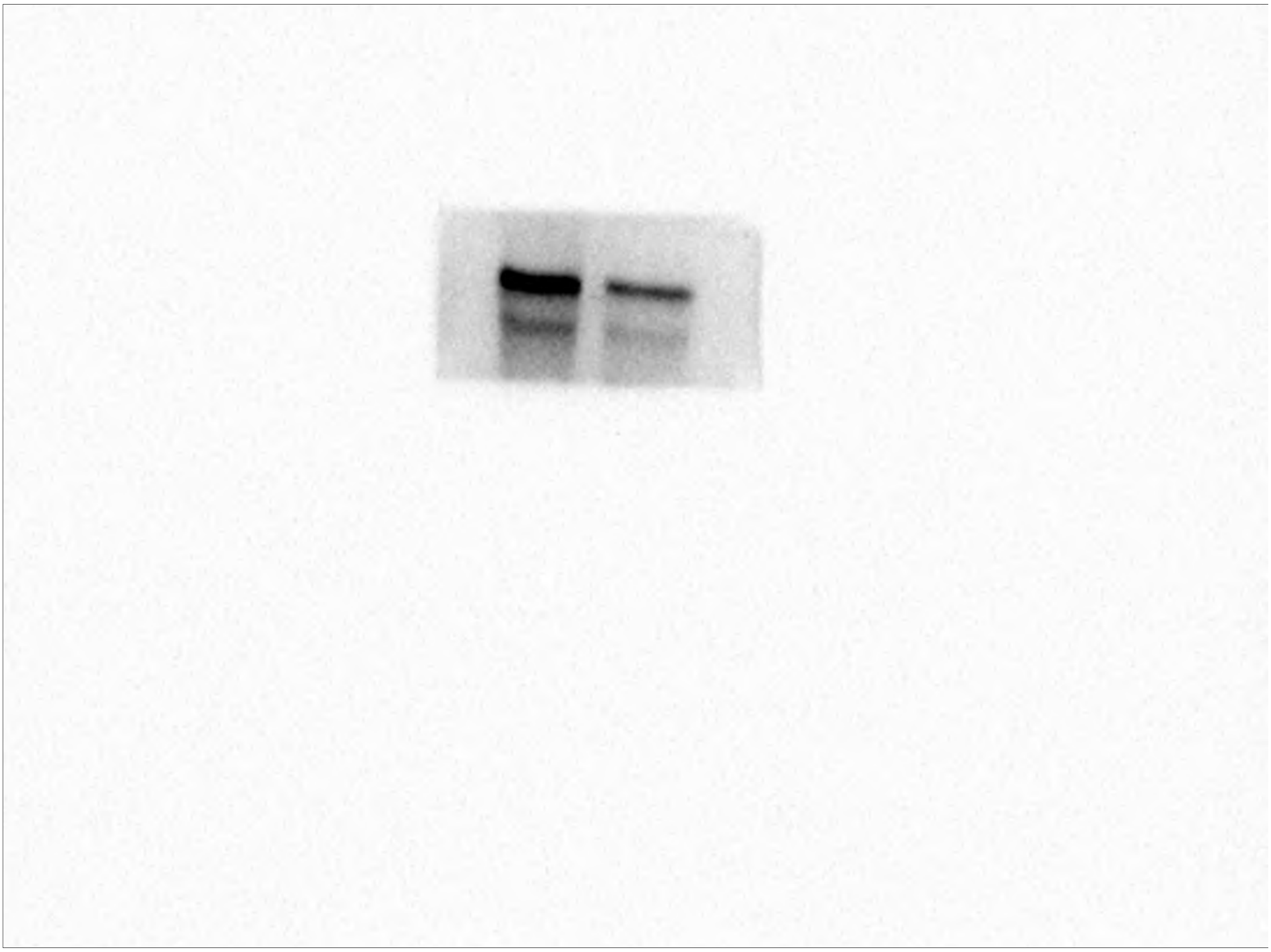

4.GAPDH1.2-sh-NC-K,sh-T-K

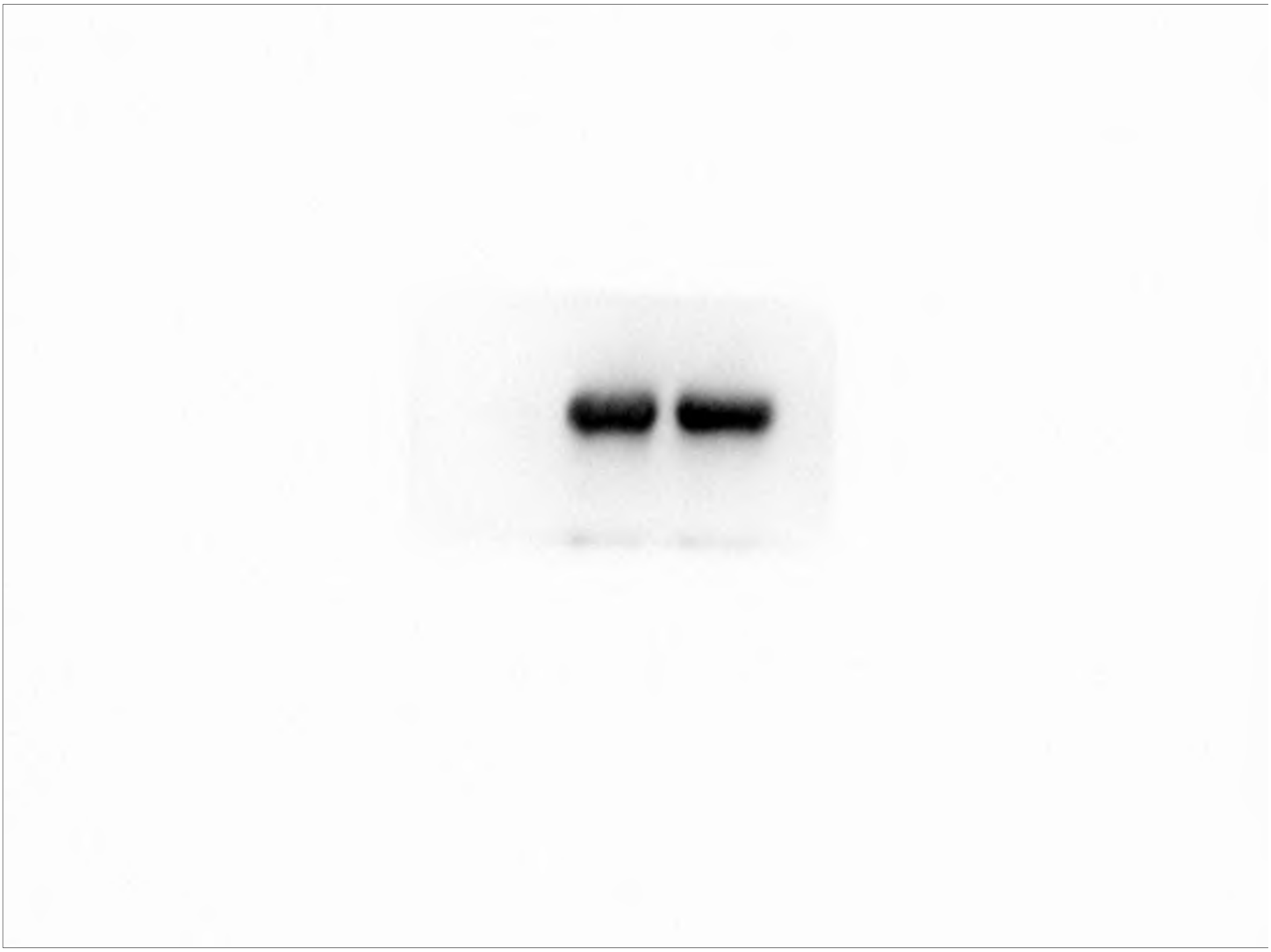

AGO2 1-supernatant，NC，circ

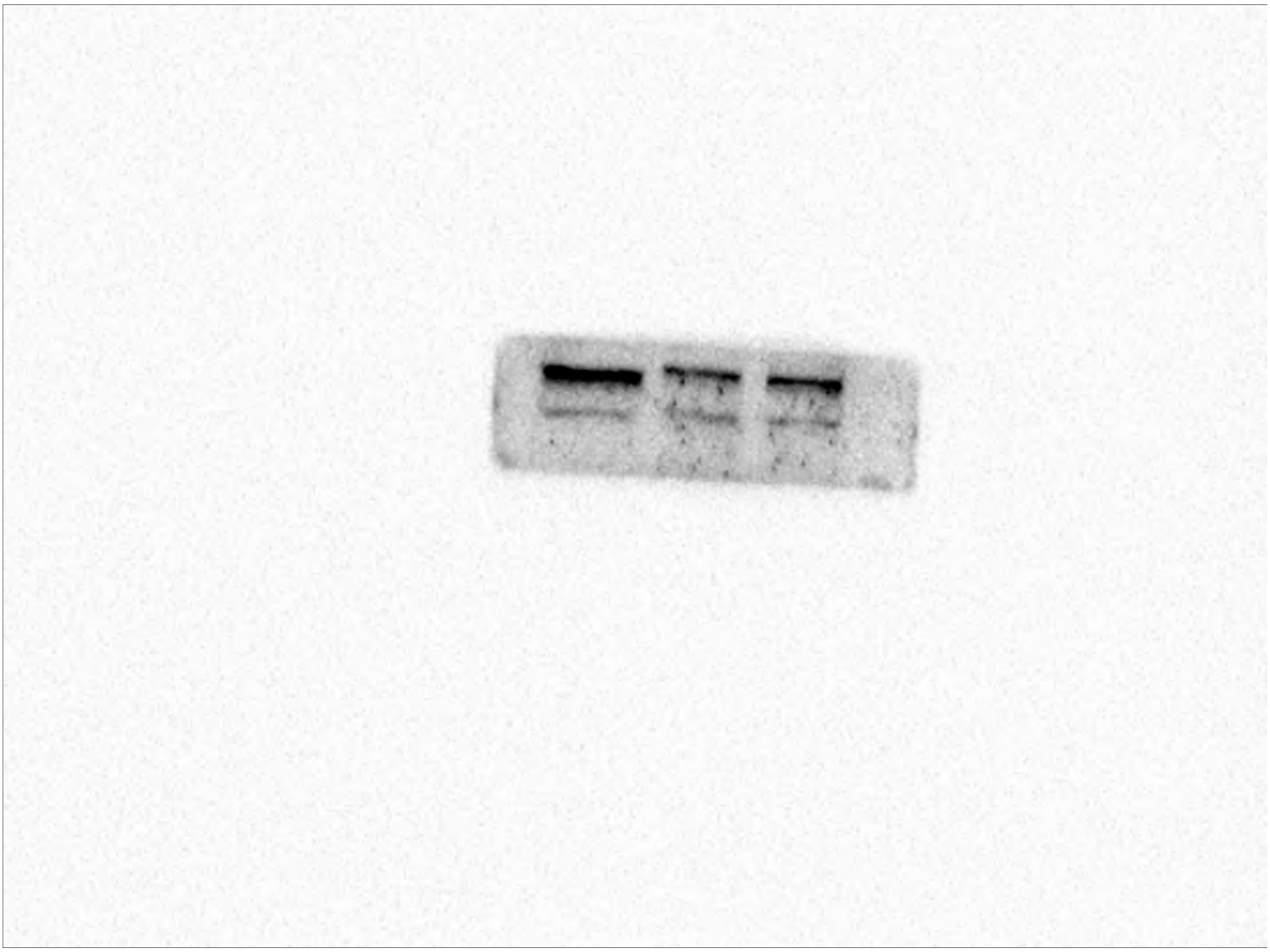

GAPDH1-supernatant，NC，circ

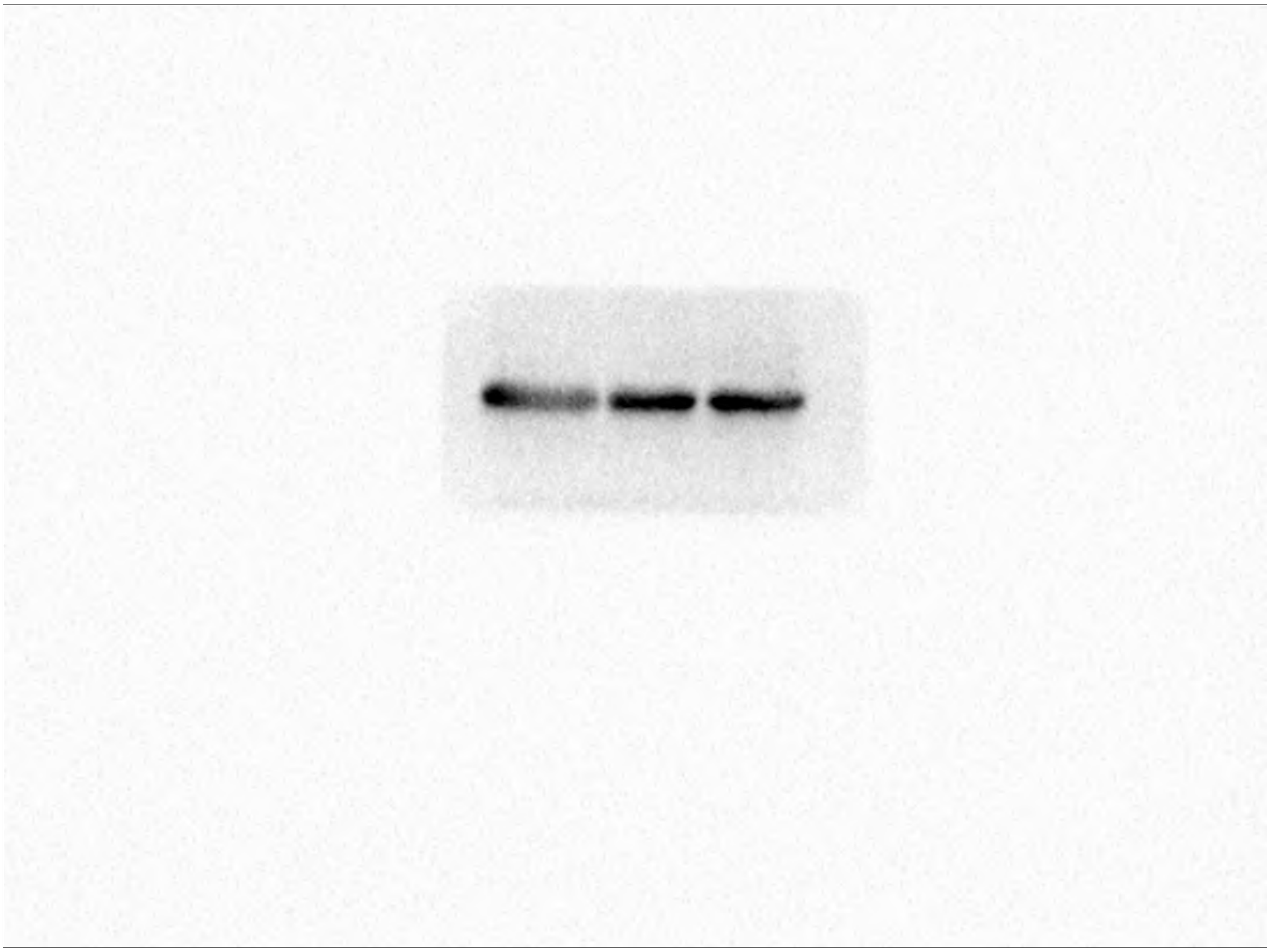

Eca3.1.2-sh-T-K-M,sh-NC-K-M

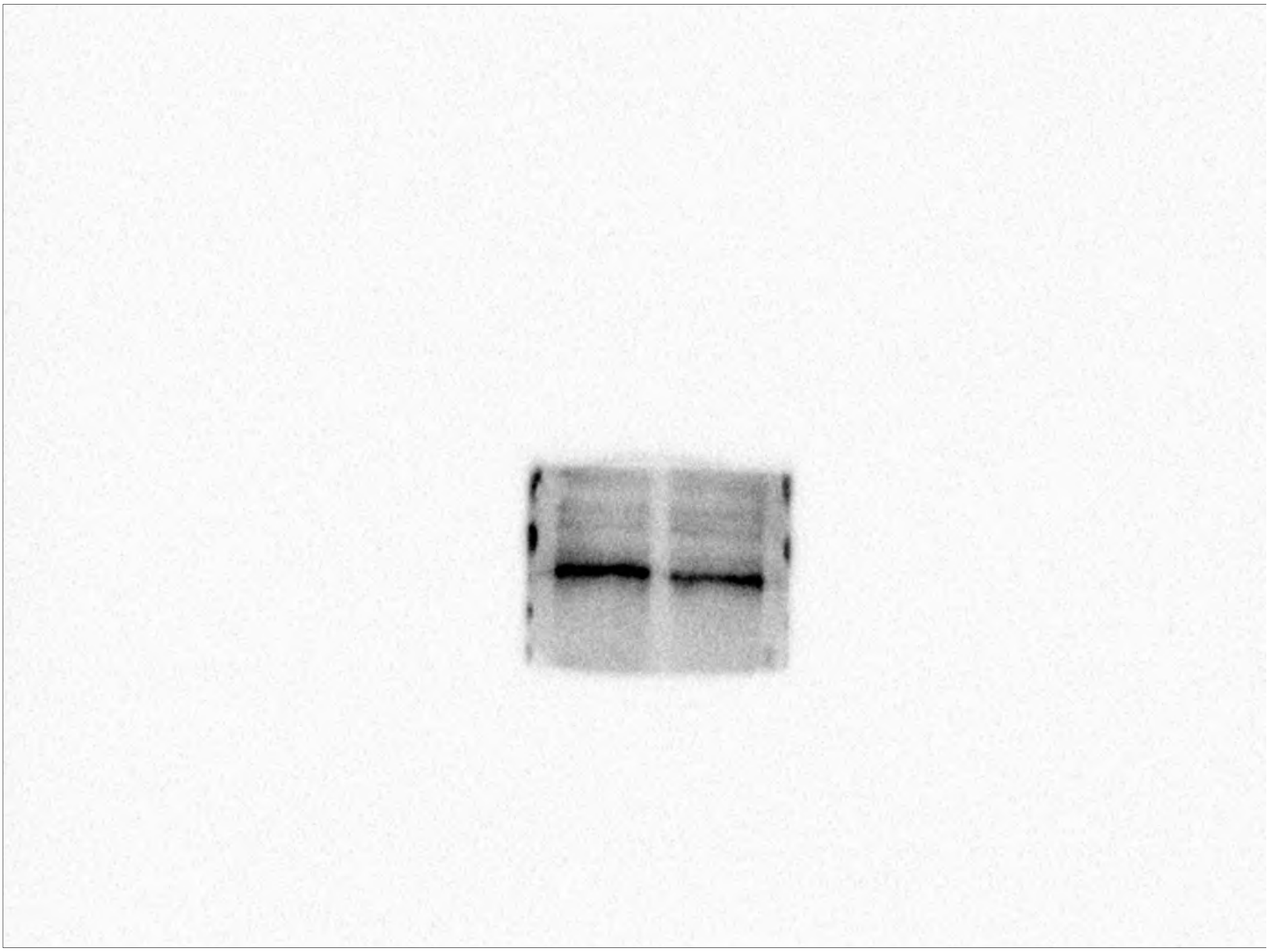

Nca5.1-sh-T-K-M,sh-NC-K-M

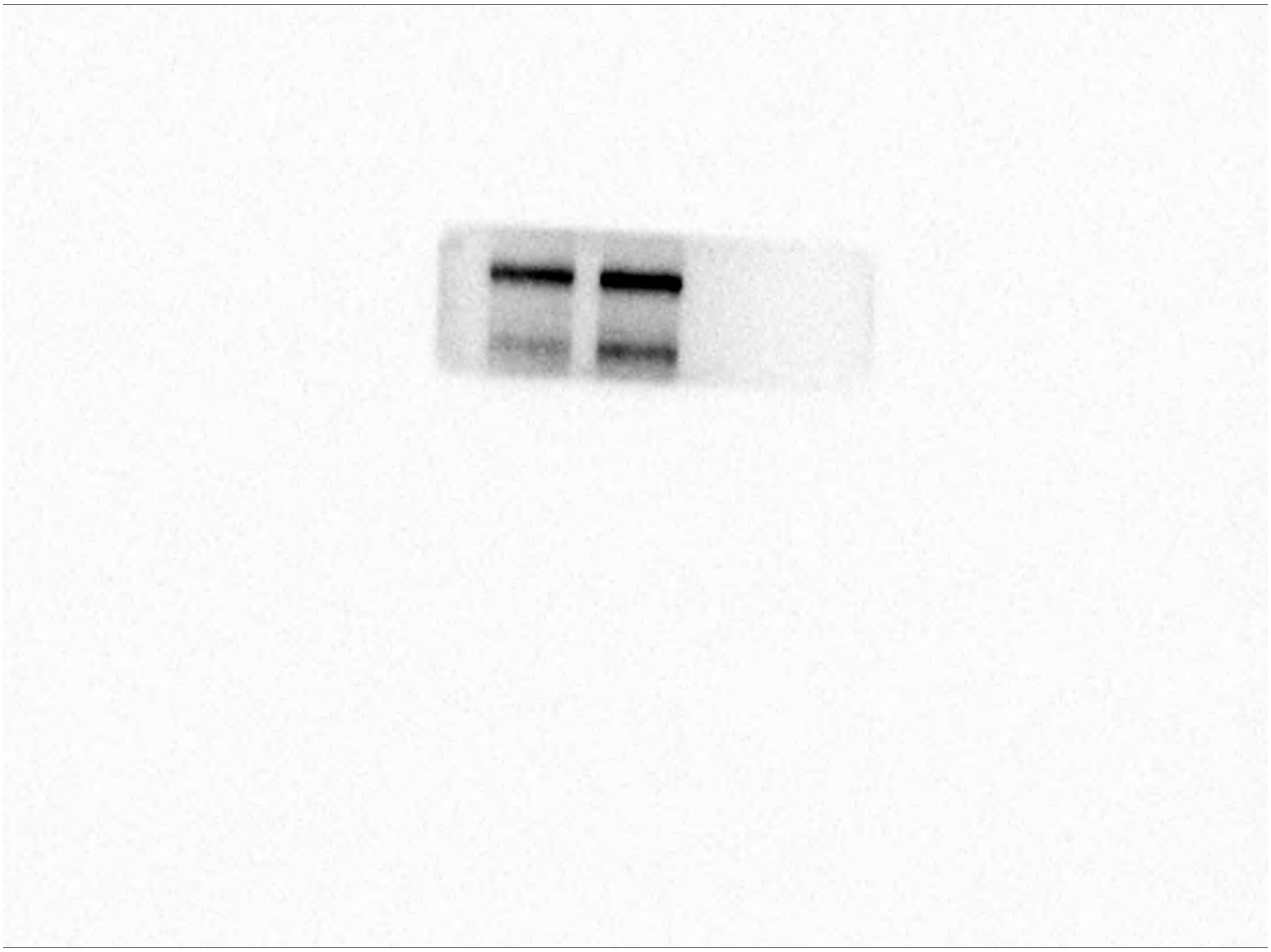

vim2.2-sh-T-K-M,sh-NC-K-M

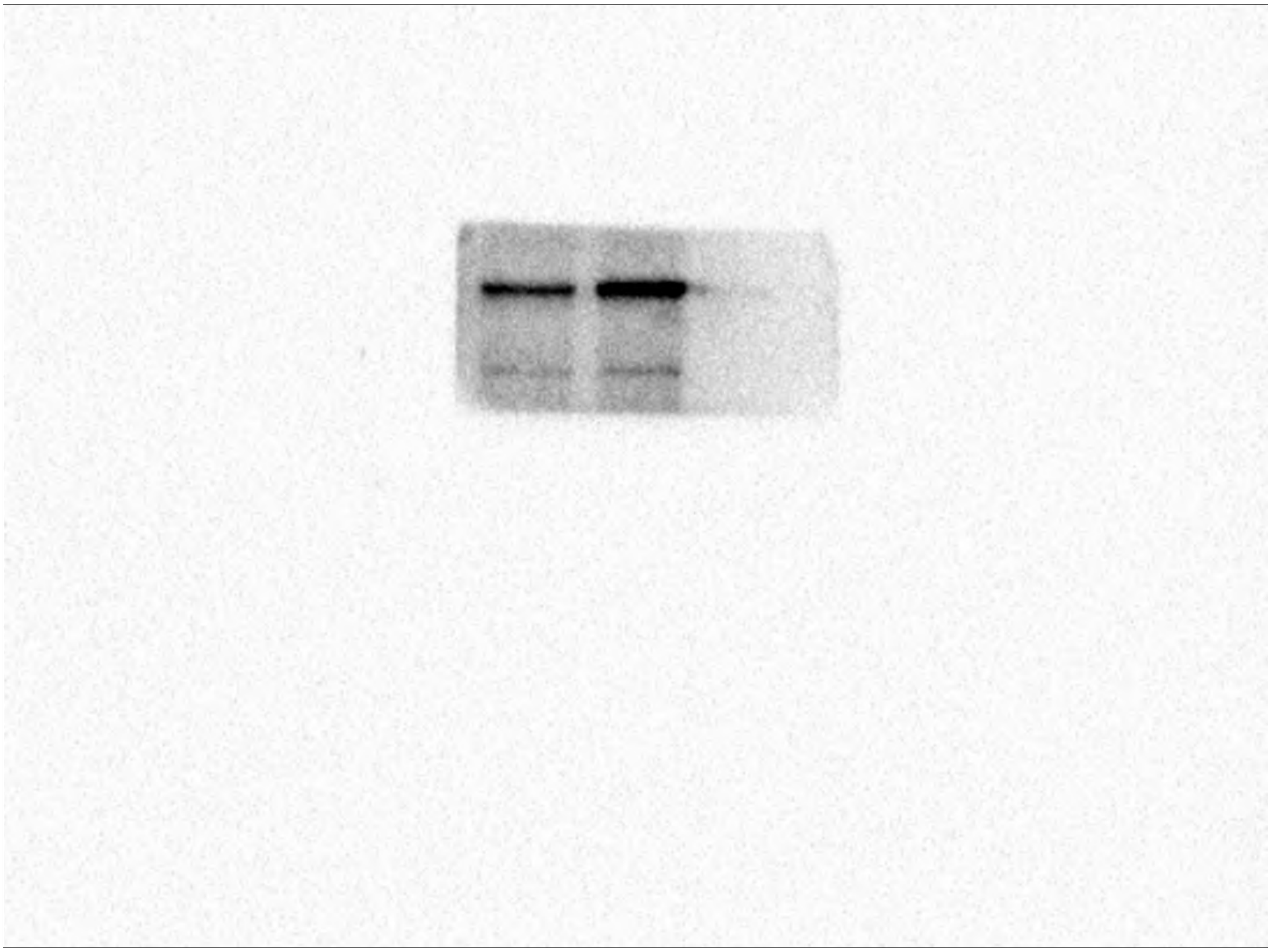

GAPDH2.3.1-sh-T-K-M,sh-NC-K-M

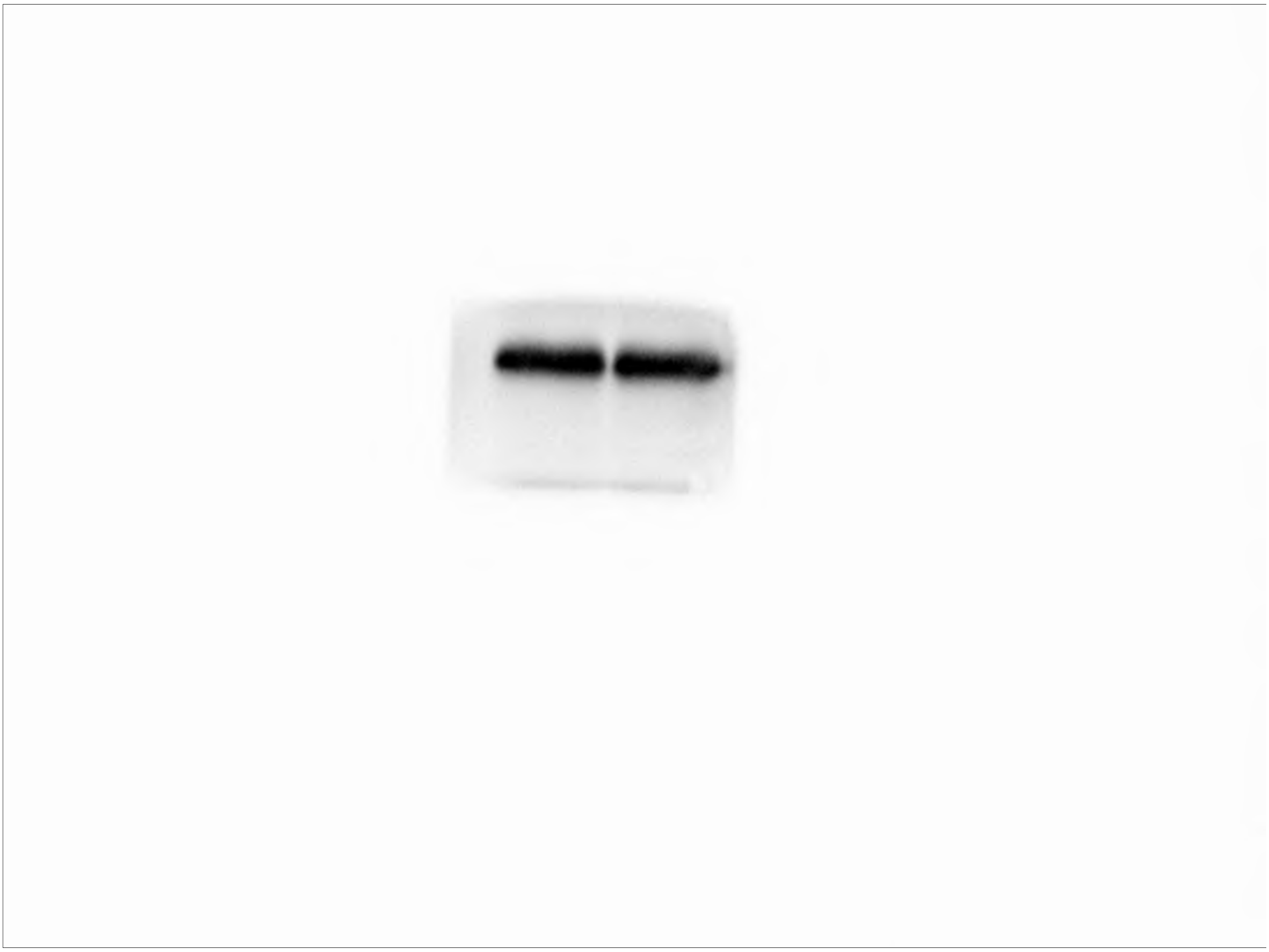

Supplement: Supplementary file 1 — original western blot [file 41420_2023_1332_MOESM1_ESM.pdf]
